# Target-triggered DNAzyme hydrogels with boron-doped atomically dispersed Fe nanozymes for one-step bacterial colorimetric sensing

**DOI:** 10.1039/d6sc07001a

**Published:** 2026-11-12

**Authors:** Tong Ji, Yanxin Li, Ruyi Zhang, Xingguo Zhai, Runluan Liu, Shenghao Xu, Xiliang Luo

**Affiliations:** a Key Laboratory of Optic-Electric Sensing and Analytical Chemistry for Life Science, MOE, College of Chemistry and Molecular Engineering, Qingdao University of Science and Technology Qingdao 266042 P. R. China xushenghao@qust.edu.cn xiliangluo@qust.edu.cn

## Abstract

Nanozyme-based bacterial detection faces two long-standing challenges: (1) dependence on multi-step enzymatic cascades that complicate assay workflows, and (2) insufficient sensitivity originating from the intrinsic low catalytic activity of nanozymes and the lack of target pre-amplification for bacterial biomarkers. To address these bottlenecks, we construct a target-triggered DNAzyme hydrogel platform integrated with boron-doped nanozymes (HCFe-B). This platform synergistically couples stimuli-responsive hydrogel-mediated controlled-release and heteroatom-doped nanozyme engineering, enabling one-step ultrasensitive bacterial detection. Upon recognition of bacterial biomarkers, the rationally designed DNAzyme hydrogel triggers one-step nanozyme release, which fully obviates the requirement for multi-step enzymatic cascades. DFT calculations verify that boron doping lowers the energy barrier of the rate-determining O–O cleavage step for atomically dispersed Fe nanozymes, greatly boosting their peroxidase-like activity. As a result, nearly 1000-fold enhancement in detection sensitivity is accomplished without any target pre-amplification. Furthermore, a convolutional neural network (CNN) machine-learning model is implemented to process the readout signals. High-accuracy blind tests on human faecal samples yield an AUC value of 98.25%, alongside 100% sensitivity and 95% specificity. These results underscore the practical promise of our platform for clinical bacterial infection prevention and public-health surveillance.

## Abstract 2

A target-triggered DNAzyme hydrogel platform integrated with catalytically enhanced boron-doped POD-specific nanozymes was proposed for one-step bacterial colorimetric sensing.

## Graphical abstract

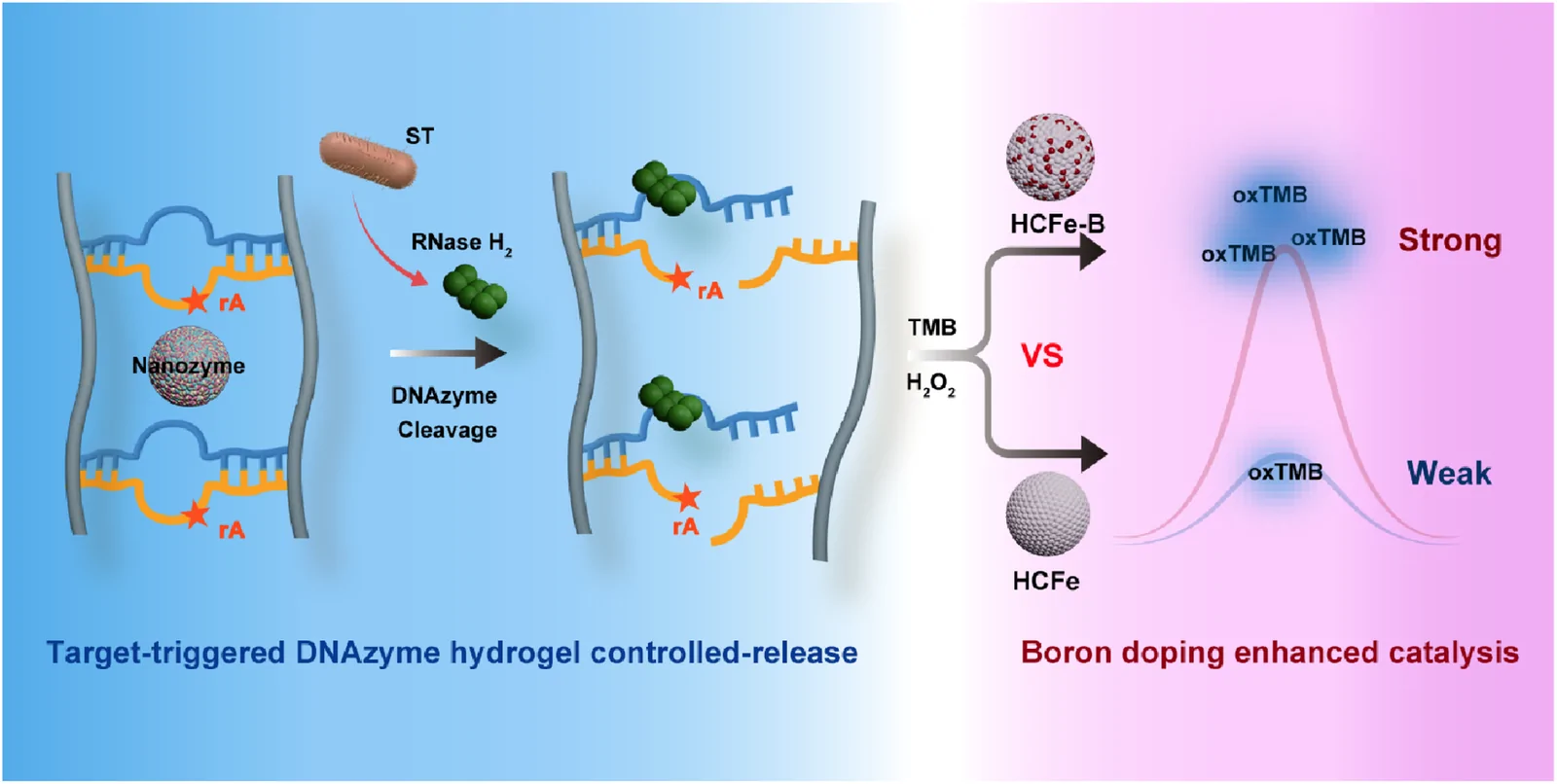

## Introduction

Developing colorimetric point-of-care diagnostics that can rapidly and accurately identify bacteria is critically important for early disease diagnosis, pathogen surveillance, and risk prediction.^1–3^ Benefiting from the unique advantages of tunable catalytic activity and excellent structural stability, nanozymes have shown great promise in high-sensitivity colorimetric analysis and have enabled transformative applications in precise point-of-care detection of pathogenic bacteria.^4–10^ Unfortunately, most current nanozymes exhibit multiple enzyme-mimicking activities simultaneously, which inevitably induces uncontrollable cross-interference and severely compromises detection accuracy.^11,12^ As an alternative, peroxidase (POD)-specific nanozymes can fundamentally eliminate interference from promiscuous enzymatic activities owing to their single POD catalytic pathway.^13,14^ Despite their excellent detection specificity, POD-specific nanozymes still face two non-negligible bottlenecks that limit their widespread use in point-of-care testing of bacteria: (1) conventional nanozyme-catalyzed sensing platforms typically require elaborate multi-step enzymatic cascades,^15^ which works against experimental simplification and makes the design and operation cumbersome; (2) the limited catalytic activity of POD-specific nanozymes, combined with the inability to pre-amplify bacteria,^13,16–19^ collectively results in insufficient sensitivity for trace-level detection of low-abundance bacteria. Therefore, constructing a one-step, highly sensitive nanozyme-catalyzed sensing platform for bacterial biomarkers that features enhanced catalytic performance while eliminating the need for target pre-amplification remains a formidable challenge.

Integrating target-responsive DNAzymes with hydrogel networks offers a rational strategy to circumvent complicated multi-enzyme cascade sensing protocols.^20–22^ Such hydrogels entrap colored agents within DNAzyme-crosslinked polymer networks, where gel dissolution upon target recognition generates visible color release. Inspired by this concept, encapsulating POD-specific nanozymes within DNAzyme-crosslinked hydrogel matrices to construct target-activatable integrated biosensing systems is expected to address this procedural bottleneck, as the POD-specific nanozymes can only be released upon target-induced DNAzyme cleavage, enabling one-step enzyme-catalyzed colorimetric detection. Furthermore, to resolve the sensitivity deficit arising from both the limited catalytic activity of POD-specific nanozymes and the inability to pre-amplify bacteria, heteroatom doping engineering serves as an effective strategy to modulate the electronic configuration and enrich the surface defect sites of POD nanozymes,^23–33^ thereby substantially enhancing catalytic kinetics while fully retaining the exclusive peroxidase pathway and ultimately achieving high sensitivity without target pre-amplification. Notably, although heteroatom doping can boost POD-like activity, many nanozyme-based bacterial detection workflows still require laborious antibody conjugation, blocking and washing operations, and often rely on complicated multi-enzyme cascade reactions.^34^ In parallel, reported DNAzyme hydrogel bacterial sensors generally employ colored agents (such as gold nanoparticles or fluorescent dyes) as readout tags.^21,35^ Their signal output is solely determined by the amount of reporters released upon target recognition, resulting in a lack of intrinsic signal amplification and thus limited detection sensitivity. Taken together, synergistically integrating target-responsive DNA hydrogel-controlled release designs with boron-doped catalytically enhanced nanozymes is expected to enable one-step, highly sensitive detection of bacteria with a simplified workflow and amplified colorimetric signal. To the best of our knowledge, however, this integrated synergistic strategy remains unexplored.

Herein, we developed a target-triggered DNAzyme hydrogel sensing platform integrated with catalytically enhanced boron-doped POD-specific nanozymes (HCFe-B) for colorimetric detection of bacteria. Compared with reported DNAzyme hydrogel-based and nanozyme-assisted bacterial detection systems, this integrated platform achieves a one-step bacterial response, and simultaneously realizes catalytic signal amplification by virtue of the tunable peroxidase-like activity of single-atom nanozymes, which constitutes the core innovation of the present sensing platform. In detail, (1) the target-triggered DNAzyme hydrogel was engineered to release encapsulated nanozymes in a single step upon target recognition, thereby eliminating the need for multi-step enzymatic cascades. (2) Boron doping of atomically dispersed single-atom Fe nanozymes delivered a 1000-fold improvement in detection sensitivity by enhancing the POD-like catalytic activity, without target pre-amplification. As indicated in Scheme 1, the target triggered DNAzyme hydrogel is constructed by crosslinking polyacrylamide chains with an acrydite-modified DNAzyme strand and a target-responsive acrydite-modified substrate strand, with boron-doped POD-specific nanozymes encapsulated within the network. Upon target recognition (taking *Salmonella typhimurium* (ST) as an example), the RNase H2 secreted by ST executes site-specific cleavage of the substrate strand, consequently disrupting the DNAzyme-crosslinked hydrogel network and triggering the release of the entrapped POD-specific nanozymes. The released nanozymes subsequently catalyze the oxidation of TMB in the presence of H_2_O_2_, generating a distinct blue colorimetric signal that enables one-step detection of bacteria. Here, the term “one-step” in our manuscript refers to the target-triggered recognition and activation process of the DNAzyme hydrogel, rather than the entire subsequent color development workflow. Upon exposure to target bacteria, this DNAzyme hydrogel achieves one-step target-responsive activation, eliminating the requirement for antibody modification, blocking, washing, or multi-enzyme cascade assembly that are commonly needed in many conventional nanozyme-based bacterial sensors. It's worth noting that the boron-doped POD-specific nanozymes exhibited a limit of detection (LOD) for ST that was nearly three orders of magnitude lower than that of their undoped counterparts. Thus, a target triggered DNAzyme hydrogel sensing platform integrated with catalytically enhanced boron-doped POD-specific nanozymes for one-step colorimetric detection of bacteria was engineered.

**Scheme 1:**
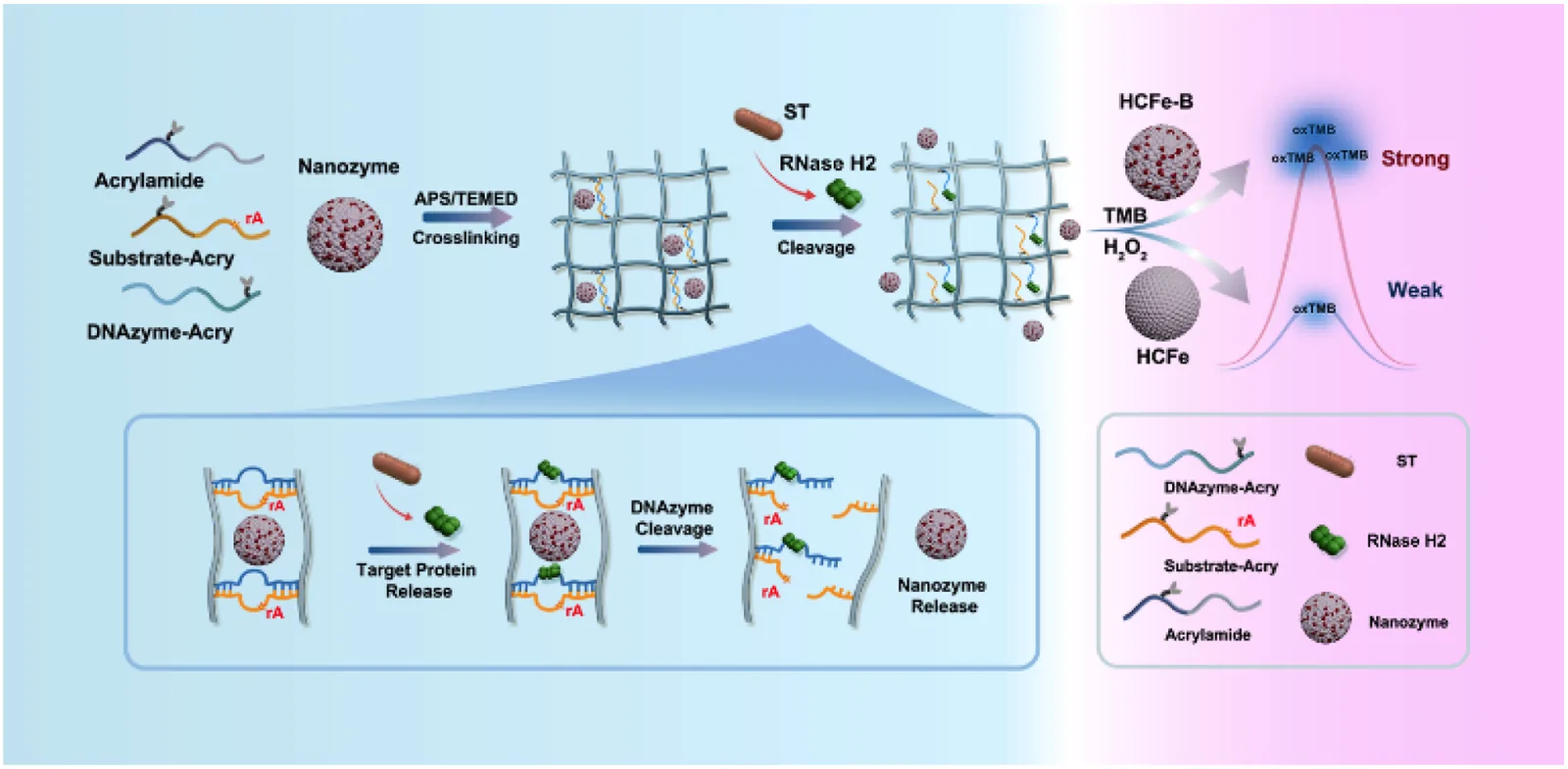
Schematic illustration of the target triggered DNAzyme hydrogels with catalytically enhanced HCFe-B for colorimetric detection of bacteria.

## Experimental

### Preparation of HCFe-B

A solution containing 0.05 mmol of hemin and 0.1 mmol of Fmoc-l-cysteine in 1 mL of DMSO was prepared. Meanwhile, 0.1 mmol of FeCl_2_·4H_2_O was dissolved in 20 mL of ice-cold ultrapure water. Under gentle stirring, the DMSO mixture was slowly introduced into the aqueous FeCl_2_ solution, followed by the addition of 0.6 mmol of NaBH_4._ The pH was then adjusted to ∼7.0, and the resulting suspension was transferred into a dialysis membrane (MWCO 8k–14k Da) for overnight dialysis against ultrapure water to eliminate residual small molecules and ions. Finally, the suspension was centrifuged for 10 min and the precipitate was re-dispersed in 5 mL of ultrapure water.

### Preparation of HCFe-B-encapsulated target-triggered DNAzyme hydrogel

30 µL of a 25% acrylamide solution, 160 µL of deionized water, 16 µL of acrydite-modified substrate strand (0.5 µM), and 16 µL of acrydite-modified DNAzyme strand (0.5 µM) were mixed and heated to 45 °C. Subsequently, 3.36 µL of APS (1.4%, w/v), 3.36 µL of TEMED (1.4%, v/v) and 40 µL of the as-prepared HCFe-B were added. After 30 min, the resulting hydrogel was washed with deionized water. Thus, the HCFe-B-encapsulated target-activated DNAzyme hydrogel was obtained.

### HCFe-B-encapsulated target-triggered DNAzyme hydrogel for bacteria (ST) sensing

10 µL of serially diluted ST suspensions was added to the HCFe-B nanozyme-encapsulated target-triggered DNAzyme hydrogel and incubated at 37 °C for 2 h. Then, 10 µL of the supernatant was transferred into a TMB colorimetric system comprising 180 µL of HAc–NaAc buffer (0.1 M, pH 4.5), 1 µL of TMB and 8 µL of H_2_O_2_. The color change was monitored and the absorption spectra were recorded.

### Application of HCFe-B-encapsulated target-triggered DNAzyme hydrogel in fecal sample analysis

0.02 g of fecal matrix was dispersed in 180 µL of sterile saline and mixed with 20 µL of serially diluted ST suspensions. After thorough homogenization, a series of artificially contaminated positive fecal samples were obtained. For negative controls, 20 µL of sterile saline replaced the bacterial suspension. Then, 10 µL of each fecal sample (positive and negative) was added to the target-responsive DNAzyme hydrogel encapsulating HCFe-B and incubated at 37 °C for 2 h. The supernatant (10 µL) was then transferred into a TMB-based colorimetric system containing 180 µL of 0.1 M HAc–NaAc buffer (pH 4.5), 1 µL of TMB substrate, and 8 µL of H_2_O_2_. Then, images of the color change were taken, followed by prediction using a convolutional neural network (CNN) model.

## Results and discussion

### Morphological and structural characterization of atomically dispersed HCFe-B

TEM micrographs displayed a uniform spherical morphology of the HCFe-B (Fig. 1A and B). EDS elemental mapping verified that C, N, O, S, Fe and B were homogeneously distributed, confirming successful boron doping that was consistent with the X-ray photoelectron spectroscopy (XPS) results (Fig. S1A and B). The high-resolution Fe 2p spectrum of HCFe-B shown in Fig. S1C revealed an obvious increase in Fe^2+^ peak area from 16.2% to 21.9%, accompanied by a decreased in Fe^3+^ peak area from 55.6% to 46.4%. This Fe^2+^ enrichment reflects higher electron density and a slightly reduced oxidation state of iron, which is attributed to boron substitution at a nitrogen site that enhances electron donation to the Fe center owing to boron's lower electronegativity. Additionally, a distinct B 1s peak was observed for HCFe-B but was absent in HCFe (Fig. S1D), confirming successful boron doping. X-ray diffraction (XRD) patterns (Fig. S2) only displayed a broad hump between 20° and 30° originating from amorphous carbon with no reflections from metallic iron or iron compounds, the results of which were in accordance with the high-resolution TEM image (no obvious lattice fringes, Fig. 1D) and the selected-area electron diffraction (SAED, Fig. 1E). Moreover, isolated bright spots can be observed using aberration-corrected high-angle annular dark-field scanning transmission electron microscopy (AC HAADF-STEM), indicating atomically dispersed single Fe atoms within the HCFe-B (Fig. 1F and G). In addition, the dispersion and coordination environment of Fe were further characterized by X-ray absorption fine structure (XAFS) spectroscopy. The Fe K-edge X-ray absorption near-edge structure (XANES) spectrum of HCFe-B nearly completely overlapped with that of Fe_2_O_3_, indicating the Fe oxidation state and similar Fe-centered local electronic environments (Fig. 1H). It's worth noting that smooth and orderly *k*^2^-weighted *χ*(*k*) oscillations were recorded throughout the entire *k* space (Fig. 1I), guaranteeing reliable fitting results in the following EXAFS quantitative analysis. Fourier transformation of the *k*^2^-weighted EXAFS data yielded the *R*-space spectra presented in Fig. 1J, from which quantitative EXAFS analysis was performed. The close agreement between the fitted curves and the raw data for HCFe-B and the reference samples in both *k*- and *R*-space (Fig. 1K, L and S3) validated the robustness of the fitting results. To gain deeper insight into the atomic distribution and to identify any minor species that may be obscured in conventional EXAFS fitting, wavelet transform (WT) analysis was conducted. As shown in Fig. 1M–P and S4, the WT analysis revealed that HCFe-B was predominantly composed of atomically dispersed Fe sites with Fe–O/B and Fe–S coordination, accompanied by a negligible fraction of sub-nanometer Fe aggregates. Taken together, these data corroborate that Fe sites in HCFe-B are dominated by atomically dispersed single-atom species.

**Fig. 1:**
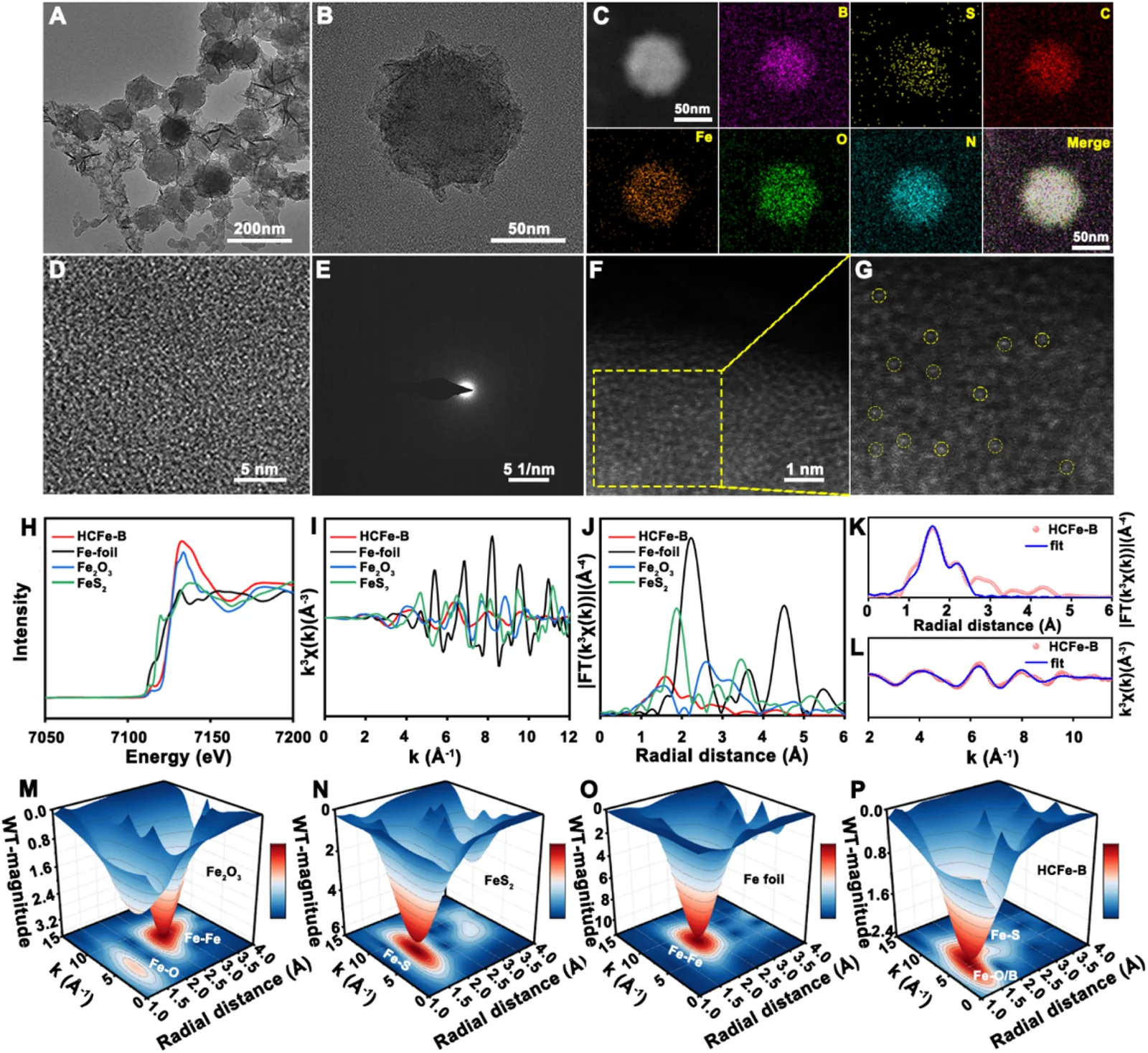
TEM images of (A) HCFe-B and (B) a single HCFe-B. (C) HAADF-STEM image together with the corresponding EDS elemental mapping. (D) HRTEM image of HCFe-B. (E) Selected-area electron diffraction pattern. (F and G) Aberration-corrected HAADF-STEM image of HCFe-B. (H) Normalized Fe K-edge XANES spectra collected for HCFe-B, together with reference samples including Fe foil, Fe_2_O_3_, and FeS_2._ Fourier-transformed (FT) EXAFS spectra in *k*-space (I) and *R*-space (J) of the Fe K-edge for HCFe-B, Fe-foil, Fe_2_O_3_ and FeS_2_. EXAFS fitting results of HCFe-B in (K) the *R*-space and (L) *k*-space. 3D wavelet transform (WT) images of the Fe K-edge from (M) Fe_2_O_3_, (N) FeS_2_, (O) Fe-foil and (P) HCFe-B.

### Investigation of boron doping on catalytic performance

Subsequently, the effect of B-doping on the catalytic performance of the nanozyme was investigated. As shown in Fig. 2A and B, the ˙OH generated from H_2_O_2_ catalyzed by HCFe-B induced a distinct blue fluorescence for terephthalic acid (TA) and a clear blue color for oxidized TMB, confirming the pronounced POD-like activity of HCFe-B. Furthermore, as illustrated in Fig. 2C, D and S5, HCFe-B exhibited almost no OXD-like or CAT-like activity across various pH ranges, demonstrating exclusive POD-specific catalytic activity under acidic conditions. Notably, B-doping was found to significantly enhance the POD-like catalytic activity (Fig. 2E). Compared to HCFe (without B-doping), HCFe-B catalyzed H_2_O_2_ to generate a higher fluorescence intensity from TA and a stronger absorbance at 652 nm from oxidized TMB (Fig. 2F). Cyclic voltammetry analysis revealed that HCFe-B produced a much higher current intensity than HCFe in the presence of H_2_O_2_ and TMB (Fig. 2G). Electron paramagnetic resonance (EPR) confirmed that HCFe-B exhibited markedly stronger quartet signals compared to HCFe, suggesting a higher production of ˙OH during the catalytic reaction.^36,37^ Steady-state kinetic studies indicated that the catalytic behavior of both HCFe and HCFe-B followed the classic Michaelis–Menten model. As shown in Fig. 2I and J, HCFe-B displayed lower *K*_m_ values for H_2_O_2_ and TMB (74.99 mM and 0.05817 mM, respectively) and a higher *V*_max_ (77.12 mM min^−1^ and 172.1 mM min^−1^, respectively), indicating its superior affinity for the substrates and significantly enhanced catalytic efficiency compared to HCFe (Fig. S6). The specific activity assay presented in Fig. 2K demonstrated a linear relationship between enzyme activity and mass, revealing that the specific activity of HCFe-B was approximately 5.93-fold higher than that of HCFe. Taken together, all of these results demonstrate that B doping can substantially enhance the catalytic activity of the nanozyme. In addition, the B doping amount was optimized (Fig. S7). Notably, HCFe-B retained excellent stability and catalytic performance after storage at different temperatures and even after one year (catalytic activity still remained at approximately 98% after being stored for one year, Fig. S8).

**Fig. 2:**
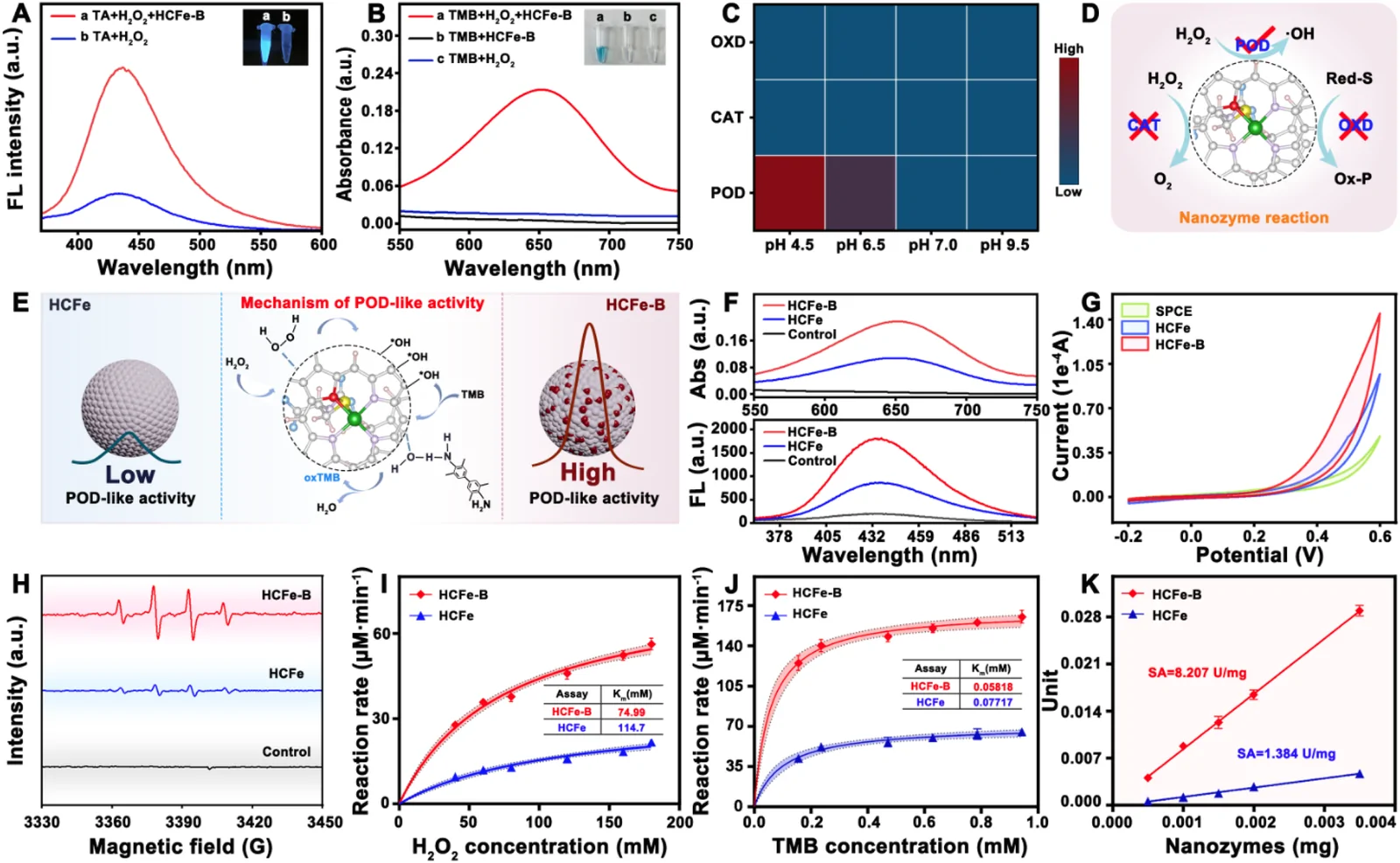
(A) Fluorescence spectra of TA and H_2_O_2_ in the (a) presence and (b) absence of HCFe-B, respectively. (B) Absorption spectra of (a) TMB + H_2_O_2_ + HCFe-B, (b) TMB + HCFe-B and (c) TMB + H_2_O_2_, respectively. (C) Comparison of OXD-like, CAT-like, and POD-like activities under different pH conditions. (D) Schematic illustration of the POD-specific HCFe-B nanozyme. (E) Schematic comparison of the catalytic activities of HCFe nanozymes before and after B doping. (F) Absorption spectra of the TMB chromogenic reaction (top) and fluorescence emission spectra of TA in the presence of H_2_O_2_ (bottom), catalyzed by HCFe-B and HCFe, respectively. (G) CV profiles of TMB and H_2_O_2_ in the presence of HCFe-B and HCFe, respectively. (H) EPR spectra of ˙OH trapped by DMPO in HCFe-B and HCFe under the same conditions. Michaelis–Menten kinetics for (I) H_2_O_2_ and (J) TMB, respectively. (K) Comparison of the POD-specific activity of HCFe-B and HCFe. SA is defined as activity units per milligram of nanozyme.

### DFT simulations of the mechanism of B-doping-enhanced POD-like activity

To further elucidate the mechanism underlying the enhanced POD-mimetic activity resulting from boron doping, density functional theory (DFT) calculations were performed. The optimized configurations of HCFe and HCFe-B are presented in Fig. 3A. Analysis of the projected density of states (PDOS) (Fig. 3B) reveals an upward shift in the d-band center of HCFe-B (−1.03 eV) relative to HCFe (−1.10 eV), which strengthens the adsorption of the *OH intermediate.^38^ As shown in Fig. 3C, the adsorption energy of H_2_O_2_ on HCFe-B (−1.077 eV) is significantly more favorable than that on HCFe (−0.378 eV). Furthermore, differential charge density analysis (Fig. 3D and S9) indicates greater electron transfer from HCFe-B to H_2_O_2_ (0.625*e*^−^) compared to HCFe (0.576*e*^−^), suggesting a stronger affinity of HCFe-B for H_2_O_2_ and highlighting the pivotal role of B in facilitating H_2_O_2_ activation.^39^ Electron localization function (ELF) maps (Fig. 3E and F) demonstrate substantial electron accumulation around the Fe center in HCFe-B, which is attributed to the electronic interaction between B and Fe.^40^ B doping reduces the oxidation state of Fe from +1.12 to +0.93, a direct consequence of boron's lower electronegativity, and this reduction promotes *OH adsorption and lowers the free-energy barrier for ˙OH generation. Finally, the free adsorption energies of the catalytic pathways and the corresponding reaction intermediates were systematically calculated. Fig. 3G and H show the free-energy profile, which divides the catalytic cycle into five steps. Step 1 is H_2_O_2_ adsorption. Step 2 is the cleavage of adsorbed H_2_O_2_ to form two *OH species, which requires energy input on the HCFe surface (energy barrier of +1.04 eV) but is exothermic on the HCFe-B surface (−0.40 eV), indicating that HCFe-B is more favorable for the initial activation of H_2_O_2_. Steps 3 and 4 involve protonation of the remaining *OH species in the acidic environment to give *H_2_O. Step 5 is the desorption of *H_2_O from the active site, which returns the model to its initial state.^41^ Over the entire reaction pathway, HCFe-B consistently exhibits lower energy barriers and more stable intermediates, confirming that boron doping significantly enhances the catalytic activity. These results also show that boron doping improves H_2_O_2_ adsorption at the active site, lowers the barrier of the rate-determining step, and ultimately facilitates efficient ˙OH generation.

**Fig. 3:**
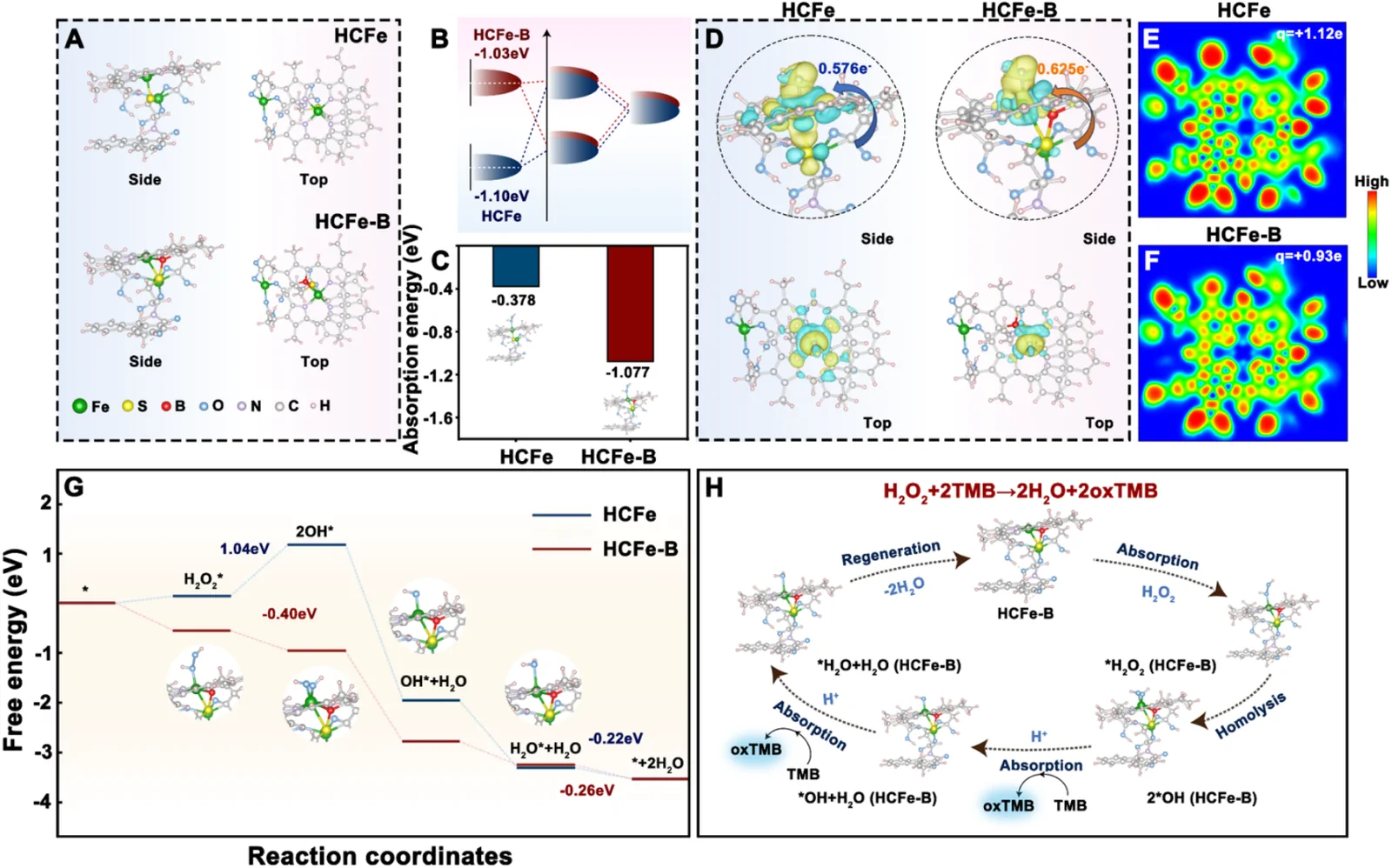
DFT insights into the B-doping-enhanced POD-like catalytic mechanism of HCFe-B. (A) Optimized geometric structures of HCFe and HCFe-B, shown in top and side views. (B) Projected density of states (PDOS) for HCFe and HCFe-B. (C) Adsorption energies of H_2_O_2_ on the surfaces of HCFe and HCFe-B. (D) Top and side views of the differential charge density for HCFe and HCFe-B, where yellow and cyan isosurfaces denote electron accumulation and depletion, respectively. (E and F) Electron localization function (ELF) contours of HCFe and HCFe-B, respectively. (G) Free-energy diagrams for the POD-like catalytic pathways of HCFe and HCFe-B, with adsorbed intermediates indicated. (H) Schematic depiction of the POD-like catalytic cycle of HCFe-B.

### Characterization of the DNAzyme hydrogel and its sensing performance

Fig. 4A and S10 illustrate the structural design of the target-triggered DNAzyme hydrogel encapsulating HCFe-B. This hydrogel was fabricated by crosslinking functional DNA sequences (acrydite-modified DNAzyme strand and target-responsive acrydite-modified substrate strand) onto an acrylamide backbone, with simultaneous encapsulation of HCFe-B nanozyme. The SEM image in Fig. 4B reveals a porous morphology of the target-triggered DNAzyme hydrogel. Energy-dispersive X-ray spectroscopy (EDS) elemental mapping (Fig. 4C) confirms the homogeneous distribution of C, N, O, S, Fe, P, and B throughout the hydrogel, verifying successful incorporation of the HCFe-B nanozyme. Rheological analysis shows that the storage modulus (*G*′) of the DNAzyme hydrogel is substantially higher than the loss modulus (*G*″), with *G*′ dominating across the entire frequency range, indicative of a loose solid-like character and good mechanical stability (Fig. 4D). Only at a strain of 1000% did *G*′ intersect with *G*″, signaling a transition to fluid-like behavior (Fig. S11). Furthermore, a hydrogel lacking functional DNA sequences was also prepared as a control to confirm the essential role of the target-triggered DNAzyme in specific target recognition. The SEM image in Fig. 4E shows that this control hydrogel likewise exhibits a porous structure. After staining with 4S Red, the DNAzyme hydrogel displayed a distinct red fluorescence under UV light, whereas the control hydrogel showed no fluorescence (Fig. 4F). Confocal laser scanning microscopy (CLSM) further revealed a network structure formed by DNA crosslinking within the DNAzyme hydrogel (Fig. 4G), which was absent in the control hydrogel (Fig. 4H). The specific recognition response of the nanozyme-encapsulated DNAzyme hydrogel toward target bacteria was then evaluated. As depicted in Fig. 4J, complementary hybridization between the FAM-labeled DNAzyme strand and the BHQ-1-labeled substrate strand quenches the FAM fluorescence. In the presence of ST, RNase H2 secreted by the pathogen mediates site-specific cleavage of the substrate strand, separating FAM from the BHQ-1 along with the fluorescence recovery (Fig. 4K) and confirming the critical role of the target-triggered DNAzyme in selective target recognition. As shown in Fig. 4I, the target-triggered DNAzyme hydrogel encapsulating HCFe-B successfully discriminates ST-positive samples (blue color), whereas the control hydrogel remained colorless, confirming the essential role of the target-triggered DNAzyme in specific target recognition. Notably, owing to the enhanced catalytic performance imparted by boron doping, the target-triggered DNAzyme hydrogel sensor encapsulating HCFe-B detects ST at concentrations as low as 10^1^ CFU mL^−1^, representing an improvement of nearly three orders of magnitude in the limit of detection compared to the sensor based on HCFe without boron doping, which reaches only 10^4^ CFU mL^−1^ (Fig. 4L and M). In addition, the absorbance signal exhibits satisfactory linear correlation with the logarithmic concentration of ST in the range from 10^1^ to 10^5^ CFU mL^−1^, with the limit of detection calculated to be 9.5 CFU mL^−1^ (3*σ*/*k*, Fig. S12). Furthermore, intra-day precision was evaluated *via* three independent parallel measurements within a single day at gradient concentrations covering 10^2^–10^7^ CFU mL^−1^, while inter-day precision was verified by repeated assays over three consecutive days. As shown in Fig. S13, the corresponding RSD values demonstrate satisfactory intra-day and inter-day reproducibility of this sensing platform. Moreover, this sensing platform exhibits excellent specificity toward ST over other bacteria (Fig. 4N). Meanwhile, negligible signal response was observed in the presence of physiologically relevant interfering biomolecules commonly present in fecal matrices, including IgG, lysozyme, thrombin, BSA and ovalbumin, confirming favorable anti-interference performance of our sensing platform for ST detection (Fig. S14). In addition, the response time (Fig. S15) and optimum temperature (37 °C, Fig. S16) of the HCFe-B-encapsulated target-triggered DNAzyme hydrogel sensor for ST detection was also optimized.

**Fig. 4:**
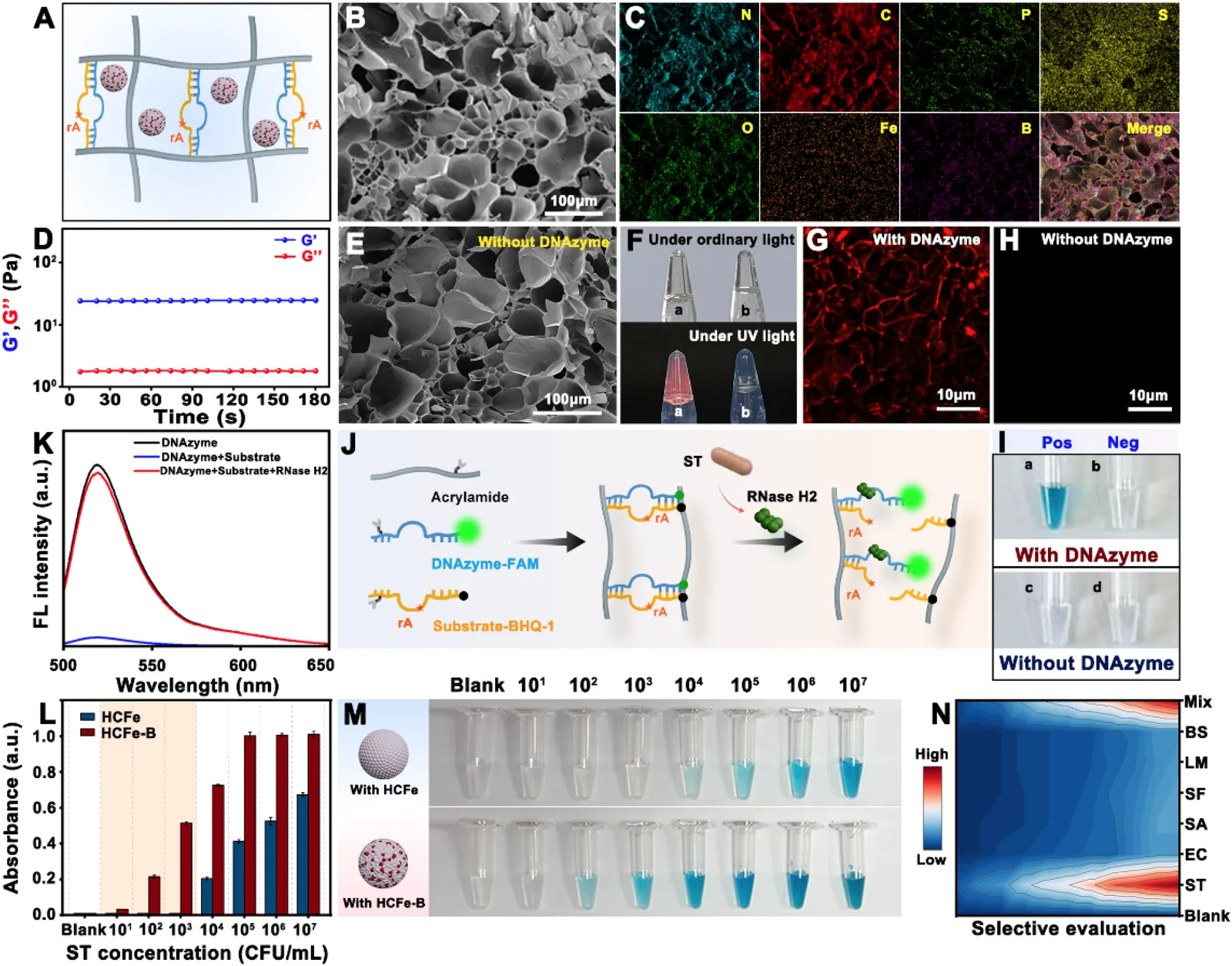
(A) Schematic illustration of the structure of HCFe-B. (B) SEM image of DNAzyme hydrogel loaded with HCFe-B. (C) EDS elemental mapping of the HCFe-B-encapsulated DNAzyme hydrogel. (D) Time-sweep rheological measurement of the DNAzyme hydrogel. (E) SEM image of the DNAzyme hydrogel without DNAzyme. (F) Photographs of the DNAzyme hydrogel and the DNAzyme-free hydrogel stained by 4S Red under ordinary (top) and UV (bottom) light. CLSM images of (G) DNAzyme hydrogel and (H) DNAzyme-free hydrogel stained by 4S Red. (I) Photographs showing the discrimination between ST-positive and ST-negative samples using the HCFe-B-encapsulated target-triggered DNAzyme hydrogel and the HCFe-B-encapsulated hydrogel lacking functional DNA sequences. (J) Schematic illustration of the specific recognition of ST by the DNAzyme hydrogel. (K) Fluorescence spectrum corresponding to panel (J). (L) Absorbance and (M) photographs of colorimetric detection of ST with different concentrations using a TMB/H_2_O_2_ catalytic system catalyzed by HCFe and HCFe-B. (N) Selectivity of the HCFe-B-based colorimetric system toward ST over other bacterial strains (all at 10^5^ CFU mL^−1^). BS: *Bacillus subtilis*; LM: *Listeria monocytogenes*; SF: *Shigella flexneri*; SA: *Staphylococcus aureus*; EC: *Escherichia coli*.

### Enhanced detection accuracy for ST by CNN-based machine learning

ST, a formidable zoonotic foodborne pathogen capable of inciting widespread outbreaks of severe gastroenteritis and sepsis, represents a serious threat to public health.^20^ Therefore, accurate and ultrasensitive detection of this pathogen is of paramount importance for preventing bacterial infections and strengthening disease control efforts. To enable precise quantification of ST, especially at extremely low concentrations, we incorporated a convolutional neural network (CNN) into the detection workflow to simultaneously boost sensitivity and classification fidelity. The blue signal produced by the chromogenic reaction was captured and processed to train the CNN model. The CNN model, by assigning prediction probabilities to each sample (ranging from 0 to 1 for each sample with values above 0.5 indicating a positive classification), enabled reliable discrimination between negative and positive cases (Fig. 5A). While both manual B/(R + G + B) ratio analysis and the CNN model achieved comparable classification accuracy for specimens exhibiting large concentration disparities between high-titer positives and negatives (Fig. 5B-(a)), the manual approach proved inadequate when confronted with low-concentration positive samples that closely resembled their negative counterparts. In contrast, the CNN model, through its capacity to extract spatial features from the acquired images, captured subtle colorimetric variations imperceptible to visual inspection and thereby delivered markedly superior classification performance (Fig. 5B-(b)). After partitioning the image dataset into training and validation subsets and subjecting the model to iterative optimization over successive epochs, the training accuracy improved substantially (Fig. 5C). Subsequently, we assessed the CNN's discriminative power using 200 samples (140 for training, 60 for testing) and found that it outperformed manual quantification, with the receiver operating characteristic curve (AUC) climbing from 64.9% to 98.25% (Fig. 5D and E).

**Fig. 5:**
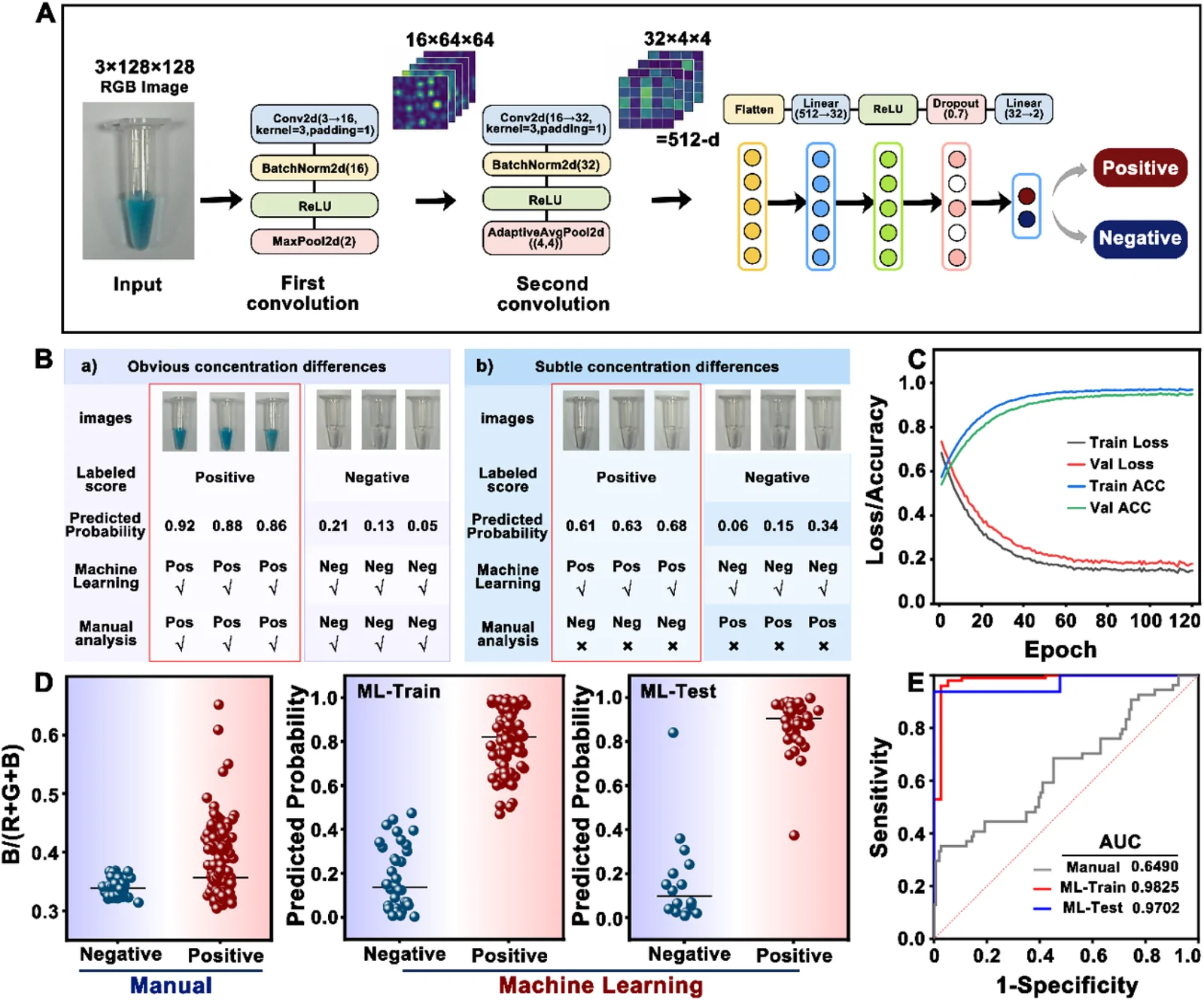
(A) CNN-based workflow for automated ST detection from smartphone-captured images. (B) CNN *versus* manual classification of ST samples with obvious (a) and subtle (b) concentration differences. (C) Training and validation loss and accuracy over 120 epochs. (D) Signal outputs: manual B/(R + G + B) ratios (left) and CNN-predicted probabilities for the training (middle) and test (right) sets. (E) ROC curves and AUC values for the training and test datasets.

### CNN-assisted ST detection in real samples

In view of the catalytically enhanced target-triggered DNAzyme hydrogel platform, its practical applicability for detecting ST in real human fecal samples was further evaluated. Fecal samples spiked with ST to yield 20 positive and 20 negative specimens were all subjected to blinded analysis using the hydrogel platform together with the CNN model (Fig. 6A), with the prediction results for these 40 blinded samples summarized in Fig. 6B–E. A comparison of the CNN outputs against the known identities of the samples revealed that every positive specimen was correctly identified while only one negative specimen was misclassified, translating into an AUC of 98.25% (Fig. 6F) together with a sensitivity of 100%, a specificity of 95%, and an overall accuracy of 97.5% (Fig. 6G). Furthermore, the results correlated strongly with those obtained from standard PCR measurements (Fig. S17), confirming the reliability of this method for ST quantification in complex fecal matrices. Therefore, these blinded validation trials establish that the platform affords high sensitivity, specificity and accuracy for ST detection in spiked fecal samples, which endows it with promising potential toward future clinical applications.

**Fig. 6:**
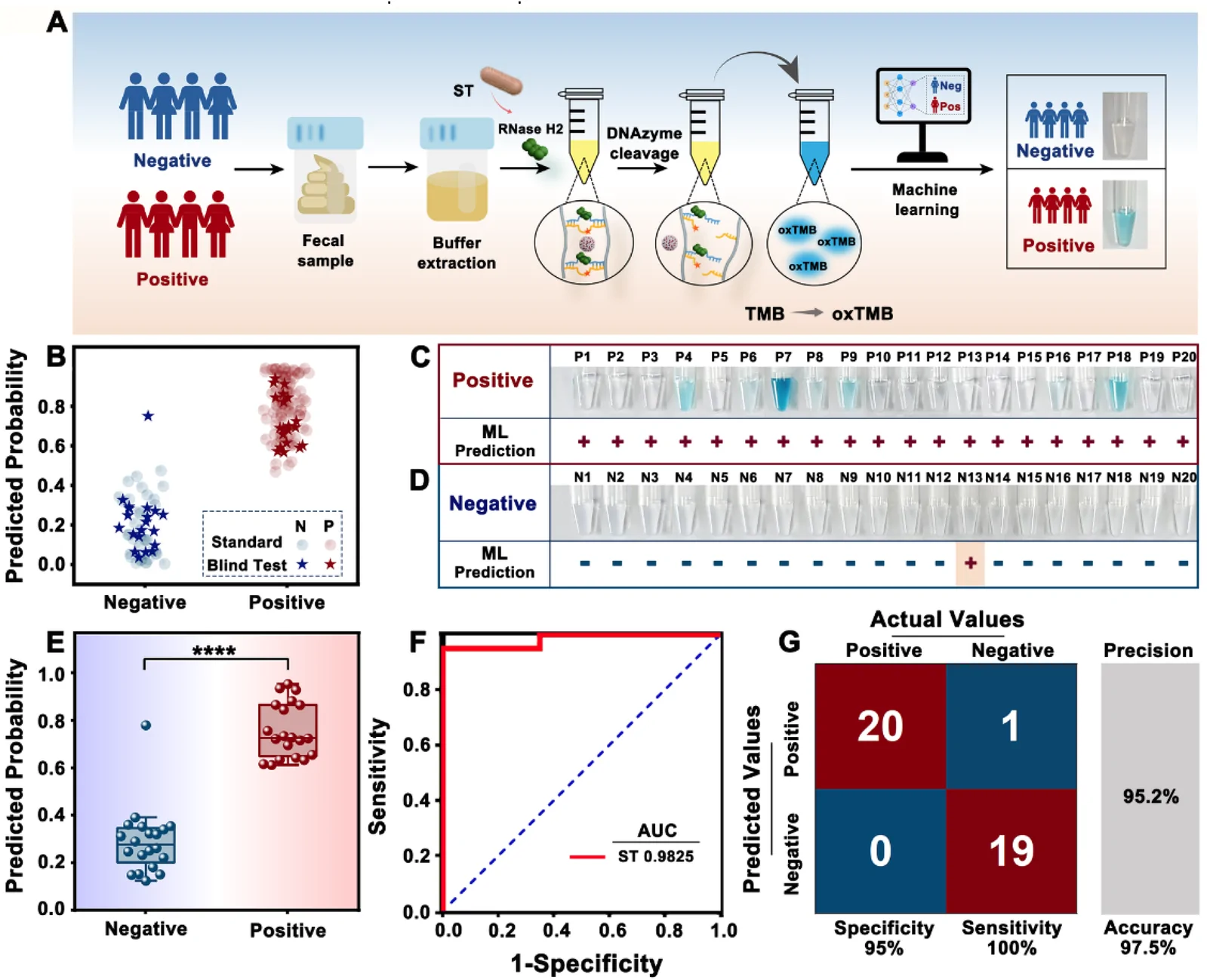
(A) Schematic illustration of the ST detection platform combining the target-triggered DNAzyme hydrogel with CNN-based analysis. (B) Blind test results for 40 unknown human fecal samples spiked with varying concentrations of ST. (C and D) Photographs showing identification of ST in human fecal samples with CNN analysis. A total of 20 positive samples (denoted as “P”) and 20 negative fecal samples (denoted as “N”) were analyzed. (E) Scatter plot comparing ST classification scores between positive and negative fecal samples using the CNN model (****, *p* < 0.0001). (F) ROC curve and AUC analysis of ST detection accuracy in human fecal samples. (G) Confusion matrix and associated classification metrics for the ST detection model evaluated on human fecal samples.

## Conclusions

In summary, a target-triggered DNAzyme hydrogel platform integrated with catalytically enhanced boron-doped POD-specific nanozymes was proposed for one-step colorimetric detection of bacteria. The target-triggered DNAzyme hydrogel enabled one-step nanozyme release upon target recognition, completely eliminating the need for multi-step enzymatic cascades. Meanwhile, XPS, EXAFS and DFT collectively demonstrated that boron doping tunes the coordination and electronic structure of atomic Fe active sites, accelerating H_2_O_2_ activation and improving POD-like catalytic activity, lowering the detection limit by approximately 1000-fold without target pre-amplification. Furthermore, by leveraging a CNN machine learning model, it was possible not only to predict positive and negative samples but also to achieve highly accurate blind testing with an AUC of 98.25%, 100% sensitivity, and 95% specificity. This work contributes valuable insights for designing platforms capable of analyzing bacteria, while also advancing the development of more efficient sensing systems for biochemical research and clinical diagnostics.

## Author contributions

T. Ji: investigation, writing – original draft. Y. X. Li: validation and formal analysis. R. Y. Zhang: formal analysis. X. G. Zhai: data curation. R. L. Liu: validation. S. H. Xu: writing – review & editing, supervision. X. L. Luo: supervision, resources, writing – review & editing.

## Conflicts of interest

The authors have declared no conflict of interest.

## Supplementary Material

SC-OLF-D6SC07001A-s001

## Data Availability

The data supporting this article have been included as part of the supplementary information (SI). Supplementary information: some experimental procedures and Tables and Figures providing additional data (materials and instruments, cyclic voltammetry test, enzymatic kinetic analysis, rheology test, DFT calculation, and so on). See DOI: https://doi.org/10.1039/d6sc07001a
