# Supplementary material for: Target-triggered DNAzyme hydrogels with boron-doped atomically dispersed Fe nanozymes for one-step bacterial colorimetric sensing
Source: Chem Sci. 2026 Nov 12. Online ahead of print. doi: 10.1039/d6sc07001a (PMC13757418; doi:10.1039/d6sc07001a)
Supplement: Supplementary file 1 [file SC-OLF-D6SC07001A-s001.pdf]

Supplementary Information (SI) for Chemical Science.
This journal is © The Royal Society of Chemistry

Supplementary Information

Target-Triggered DNAzyme Hydrogels with Boron-Doped Atomically Dispersed Fe Nanozymes for One-Step Bacterial Colorimetric Sensing

Tong Ji[a], Yanxin Li[a], Ruyi Zhang[a], Xingguo Zhai[a], Runluan Liu[a], Shenghao Xu[*a] and Xiliang Luo[*a]

a Key Laboratory of Optic-electric Sensing and Analytical Chemistry for Life Science, MOE; College of Chemistry and Molecular Engineering; State Key Laboratory of Advanced Optical Polymer and Manufacturing Technology, Qingdao University of Science and Technology, Qingdao 266042, P. R. China.

[*]Corresponding author:

Shenghao Xu (xushenghao@qust.edu.cn); Xiliang Luo (xiliangluo@qust.edu.cn) Key Laboratory of Optic-electric Sensing and Analytical Chemistry for Life Science, MOE; College of Chemistry and Molecular Engineering; State Key Laboratory of Advanced Optical Polymer and Manufacturing Technology, Qingdao University of Science and Technology, Qingdao 266042, P. R. China.

List of Contents

Materials and instruments....Page S-3

Cyclic voltammetry test....Page S-4

Determination of POD-like activity....Page S-4

Enzymatic kinetic analysis....Page S-4

Rheology test....Page S-5

Density functional theory calculation....Page S-5

Construction of convolutional neural network (CNN) model....Page S-5

Detailed DNA and RNA sequences, Table S2, Table S3....Page S-7

Fig S1 and Fig S2....Page S-8

Fig S3....Page S-9

Fig S4 and Fig S5....Page S-10

Fig S6, Fig S7 and Fig S8....Page S-11

Fig S9 and Fig S10....Page S-12

Fig S11 and Fig S12....Page S-13

Fig S13 and Fig S14....Page S-14

Fig S15 and Fig S16....Page S-15

Fig S17....Page S-16

Materials

Ammonium persulfate (APS), N, N, N',N'-tetramethyl ethylenediamine (TEMED), hemin, iron(II) chloride tetrahydrate ($FeCl_2 \cdot 4H_2O$), sodium hydroxide (NaOH), acrylamide, sodium phosphate monobasic (NaH_2PO_4), sodium borohydride ($NaBH_4$), sodium acetate anhydrous (NaAC), sodium phosphate dibasic (Na_2HPO_4), terephthalic acid (TA), 3, 3', 5, 5'-tetramethylbenzidine (TMB), dimethyl sulfoxide (DMSO), fmoc-L-cysteine, hydrogen peroxide (H_2O_2), sodium borohydride ($NaBH_4$), acetic acid (HAc) were obtained from sigma-aldrich (U.S.A.). Salmonella typhimurium (ST) was purchased from Sangon Biotech. RNase H2 was purchased from New England Biolabs (Ipswich, MA, U.S.A.). Detailed oligonucleotide sequences are shown in **Table S1**. Human fecal sample collection was performed with informed consent from healthy volunteers and was approved by the Institutional Review Board of the Affiliated Hospital of Qingdao University.

Instruments

The morphological characteristics of the DNAzyme hydrogel were examined using scanning electron microscopy (SEM) on a TESCAN MIRA LMS instrument (Czech Republic). The morphology of the HCFe-B nanozyme was characterized via transmission electron microscopy (TEM) on a Thermo Scientific Talos T200X instrument (USA). High-resolution atomic structure visualization was achieved using aberration-corrected high-angle annular dark-field scanning transmission electron microscopy (AC HAADF-STEM, Titan Cubed Themis G2300). X-ray photoelectron spectroscopy (XPS) measurements were conducted on a Thermo Fisher ESCALAB 250Xi (USA). Powder X-ray diffraction (XRD) data were obtained with a Rigaku D/MAX 2500V diffractometer. All XAFS data were measured at the Shanghai Synchrotron Radiation Facility (SSRF, China). The generation of hydroxyl radicals (•OH) was detected by electron paramagnetic resonance (EPR) using a Bruker A300 spectrometer. Ultraviolet–visible (UV–vis) absorption spectra were

recorded on a UV-2600 spectrophotometer (Shimadzu, Japan), and fluorescence spectra were measured on an F-7000 fluorescence spectrometer (Hitachi, Japan).

Cyclic voltammetry test

A mixture containing 45 μL of HAc-NaAc buffer, 0.25 μL of TMB, 2 μL of H_2O_2, and 2.5 μL of HCFe-B nanozyme were drop-cast onto the surface of a screen-printed carbon electrode (SPCE). Cyclic voltammetry (CV) measurements were then performed using an electrochemical workstation. The POD-like activity of HCFe-B nanozyme was evaluated by comparing the oxidation peak current of TMB.

Determination of POD-like activity

10 μL of HCFe-B or HCFe nanozyme was added to 180 μL of HAc-NaAc buffer (0.2 M, pH 4.5) containing 1 μL of TMB (20 mg/mL in DMSO) and 8 μL of H_2O_2 (10 M). The absorbance of oxTMB was recorded at 652 nm using a spectrophotometer.

Enzymatic kinetic analysis

To characterize the kinetics of HCFe-B and HCFe nanozymes with H_2O_2 as the substrate, H_2O_2 was added to the reaction mixture at final concentrations of 40, 60, 80, 120, 160, and 180 mM. The absorbance at 652 nm was measured to determine the initial reaction velocities at each H_2O_2 concentration. To characterize the kinetics of HCFe-B and HCFe nanozymes with TMB as the substrate, TMB was added at final concentrations of 0.15, 0.24, 0.47, 0.63, 0.79, and 0.94 mM. The absorbance at 652 nm was recorded to calculate the initial velocities at varying TMB concentrations. Michaelis–Menten kinetic parameters were determined by measuring initial reaction rates under varied substrate concentrations (TMB or H_2O_2), while keeping the other substrate at saturated concentration. Absorbance traces were recorded, and initial velocities were fitted to the classic Michaelis-Menten equation by non-linear regression using GraphPad Prism: $v = V_{max} \cdot [S]/(K_m+[S])$. All

kinetic measurements were performed with at three independent replicates (n=3). Data are presented as mean values, and error bars in figures represent standard deviation (SD). Complete kinetic parameters including V_{max}, K_m, 95 % confidence intervals (CI), coefficient of determination (R^2), and residual standard deviation ($S_{y.x}$) are summarized in **Table S2** and **Table S3**.

Rheology test

Time sweep tests were performed at 25 °C under a constant strain of 1% and a fixed frequency of 1 Hz for 3 min. Subsequently, strain sweep tests were conducted from 0.01% to 10000% strain at a constant frequency of 1 Hz to determine the linear viscoelastic region.

Density functional theory calculation

All structural optimizations were performed using density functional theory (DFT) as implemented in the Vienna Ab Initio Simulation Package (VASP), employing the projector augmented wave (PAW) method. The exchange–correlation interactions were described by the Perdew–Burke–Ernzerhof (PBE) generalized gradient approximation (GGA) functional, combined with the semi-empirical DFT-D3 correction for dispersion interactions. A plane-wave cutoff energy of 450 eV was used for all geometry relaxations. Brillouin zone sampling of the HCFe-B system was carried out with a 2×2×1 Monkhorst–Pack k-point mesh. Self-consistent field (SCF) iterations were converged to 10^{-5} eV per atom, and ionic relaxation was terminated when the maximum residual force on any atom fell below 0.02 $eV·Å^{-1}$. Spin-polarized calculations were included to properly capture the magnetic behavior of the slab models. The optimized HCFe-B model exhibits lattice parameters of a=17.00 Å, b=16.00 Å, c=15.00 Å, and α=β=γ=90.

Construction of convolutional neural network (CNN) model

A lightweight convolutional neural network was built for end-to-end binary classification of tube images into bacterial-negative and bacterial-positive categories. The network consists of two main

parts: a convolutional feature extractor and a fully connected classifier. The feature extractor contains two sequential convolutional blocks, each including a convolution operation, batch normalization, a ReLU activation, and spatial pooling. After the second block, adaptive global pooling compresses the learned feature maps into a fixed-dimensional vector, which is then fed into the classifier head. The classifier head comprises a linear projection with ReLU activation, a dropout layer for regularization, and a final linear layer that outputs two logits. Throughout the network, ReLU activation was used for all layers except the last one. A sigmoid function was applied to the output logit corresponding to the positive class to generate a risk score between 0 and 1; samples with a score $\geqslant 0.5$ were classified as high-risk (positive), otherwise as low-risk (negative). A total of 200 colorimetric images were collected under consistent imaging conditions including camera distance, exposure time and background, to avoid extra visual interference. The dataset was randomly split into a training set of 140 images and an independent test set of 60 images. To mitigate overfitting, we adopted early stopping during model training and monitored the loss and accuracy curves of both training and test sets simultaneously; training was terminated once the test-set accuracy stopped improving while the training accuracy kept rising. No image augmentation was applied here to keep the evaluation straightforward. The 60 test images were never involved in model parameter tuning, which serves as an independent cohort to verify the classification reliability of our CNN model. To address class imbalance and the scarcity of low-concentration positive samples, the training procedure incorporated several strategies: a class-weighted cross-entropy loss with label smoothing, a learning rate schedule combining a short linear warm-up with cosine annealing, and a weighted sampling mechanism that balanced the contributions of negative, low-concentration, and high-concentration groups within each mini-batch. The model was trained until convergence, and the final checkpoint was retained for inference on the test set.

Table S1 Detailed DNA and RNA sequences

Name	Sequence (5'-3')
DNAzyme	/5Acryd/GATGTGCCTTGATATTGATGGTGCATAGTCCTCC GATCCTGA/FAM
Substrate	/5Acryd/TTTTTGGAT/BHQ1CAGGACAACTGACTrATGAC CAGTTCAATATCAA

Table S2 Michaelis–Menten kinetic parameters and fitting statistics for H_2O_2

H_2O_2	HCFe-B	HCFe
V_{max}	77.12 (95% Cl: 70.70-84.88)	33.02 (95% Cl: 27.53-41.59)
K_m	74.99 (95% Cl: 59.94-93.82)	114.7 (95% Cl: 76.94-176.5)
R^2	0.9607	0.9182
$S_{y.x}$	2.080	1.271

Table S3 Michaelis–Menten kinetic parameters and fitting statistics for TMB

TMB	HCFe-B	HCFe
V_{max}	172.1 (95% Cl: 166.1-178.4)	65.66 (95% Cl: 61.61-70.15)
K_m	0.05818 (95% Cl: 0.04382-0.07412)	0.07717 (95% Cl: 0.04997-0.1101)
R^2	0.9593	0.9599
$S_{y.x}$	3.303	1.892

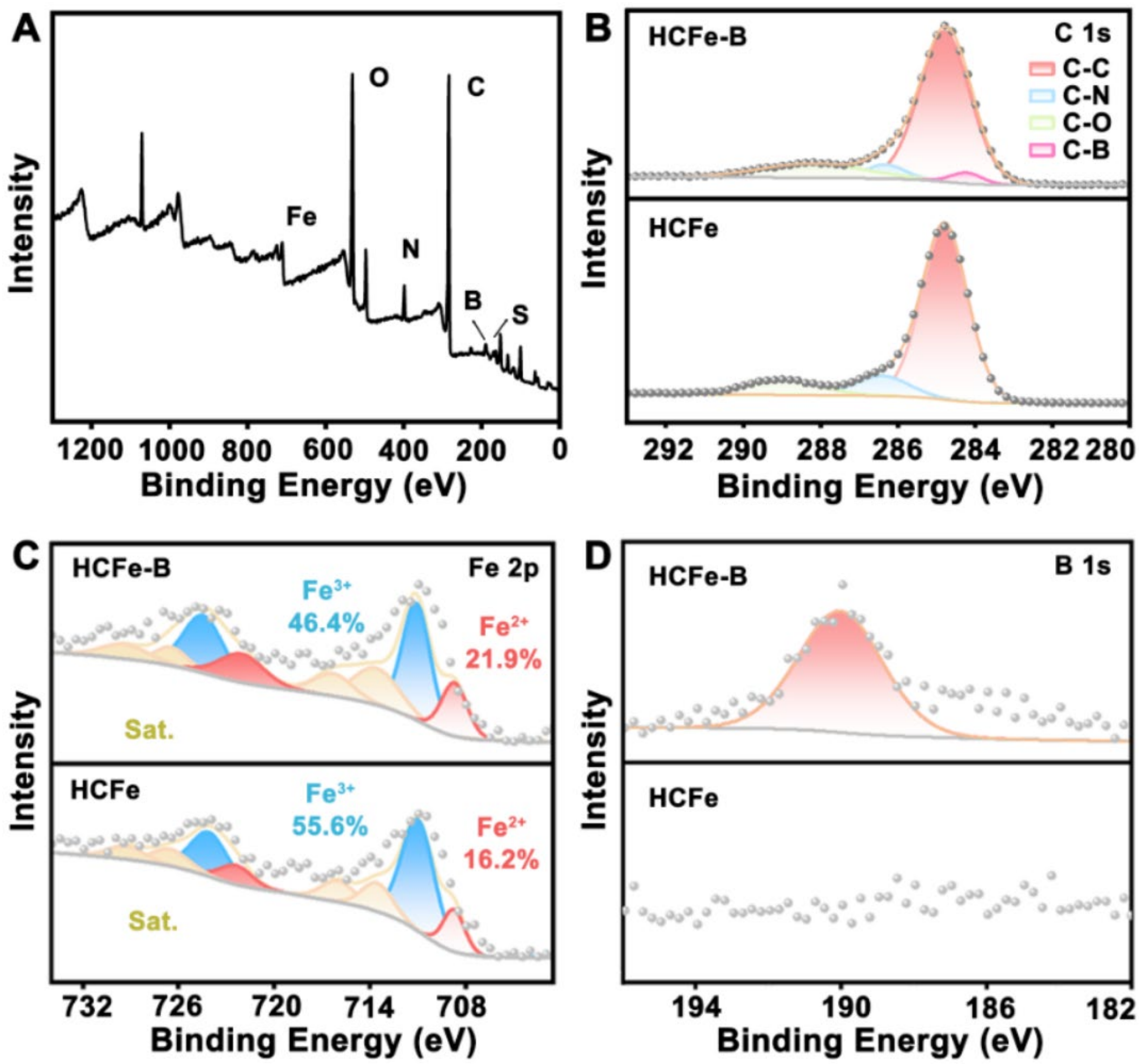

Fig S1 (A) XPS fully scanned spectrum of HCFe-B, (B) High-resolution XPS spectra of C 1s for HCFe-B and HCFe, (C) High-resolution XPS spectra of Fe 2p for HCFe-B and HCFe, (D) High-resolution XPS spectra of B 1s for HCFe-B and HCFe.

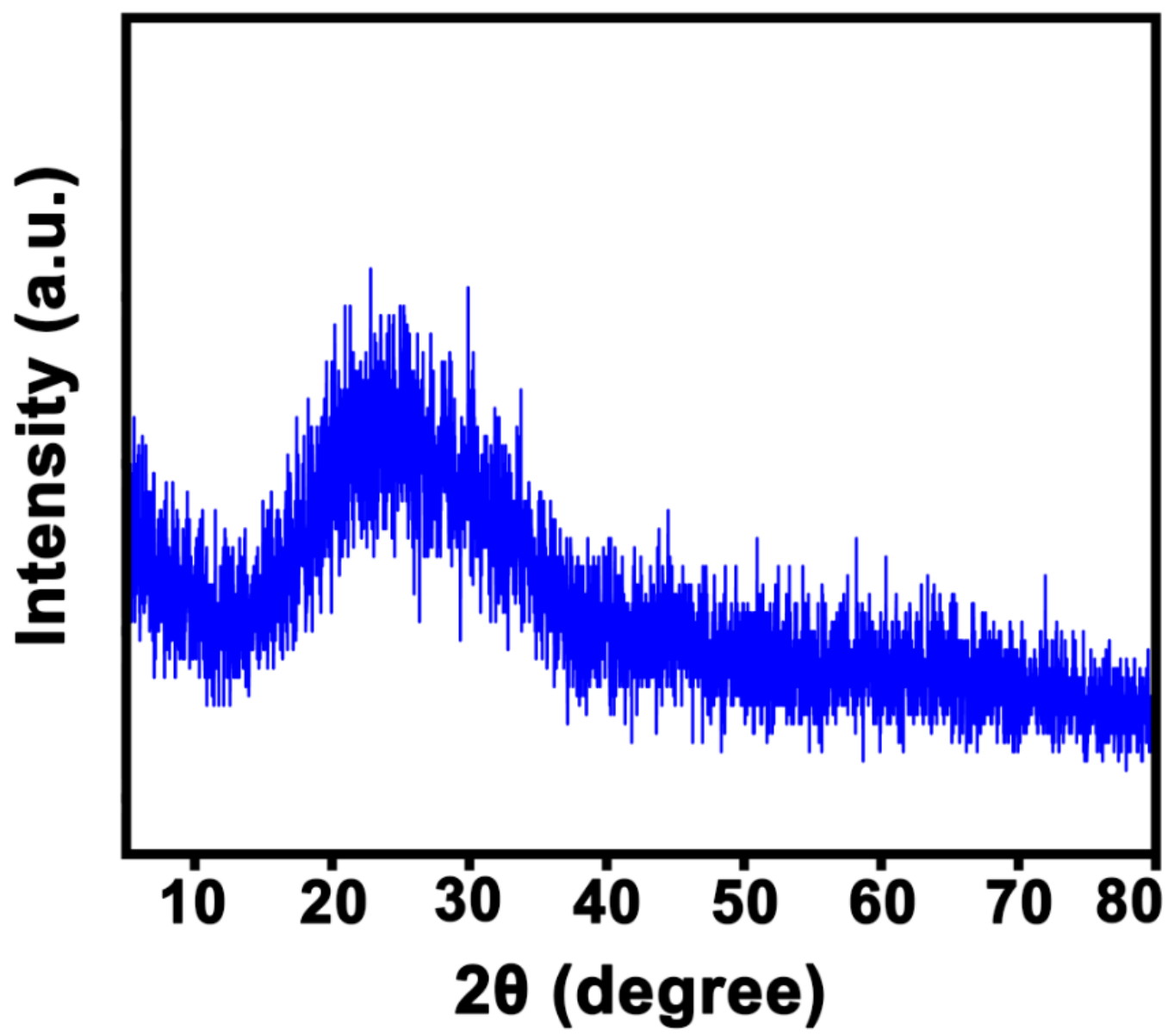

Fig S2 XRD pattern of HCFe-B.

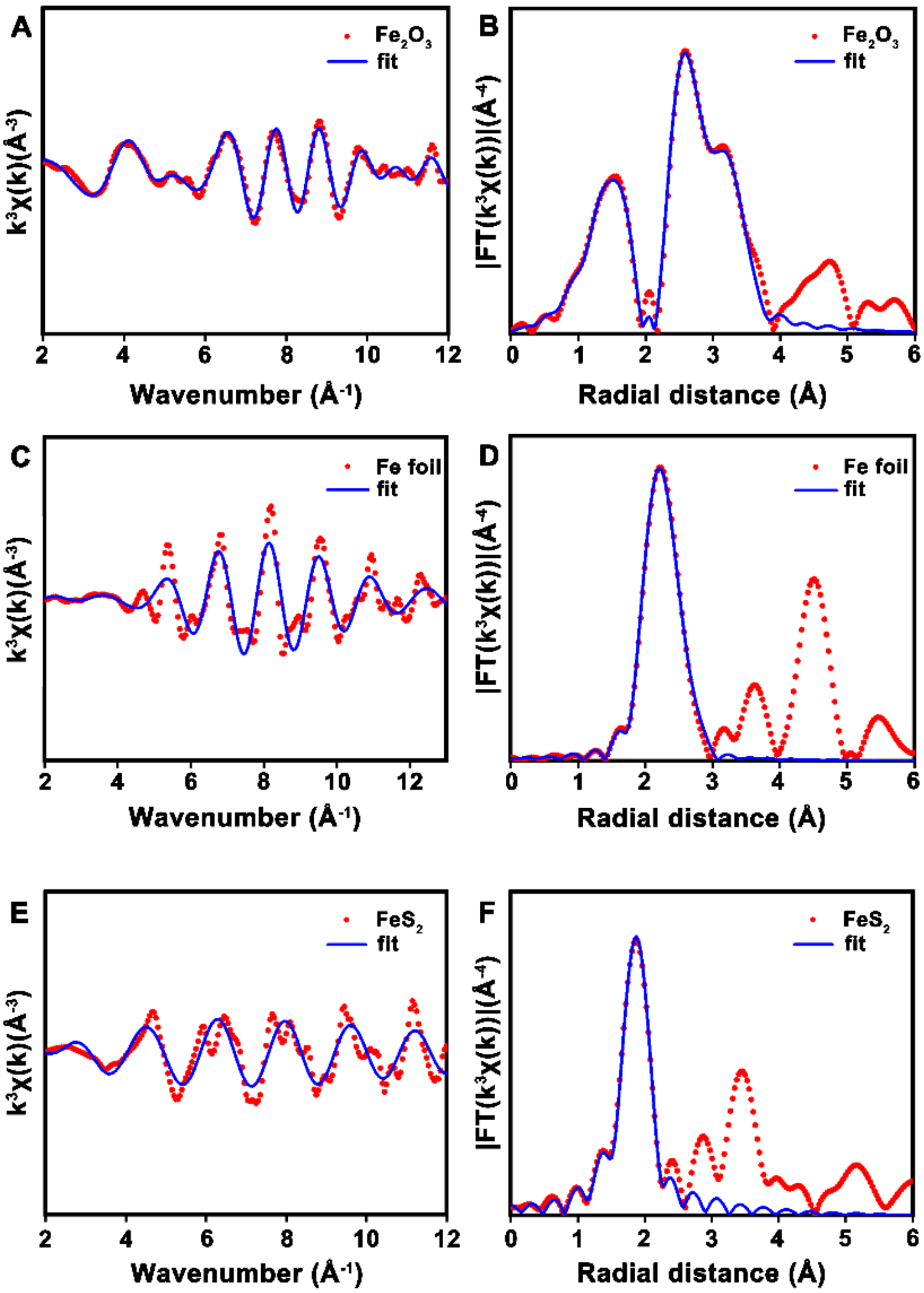

Fig S3 EXAFS fitting results of Fe foil, Fe_2O_3 and FeS_2 at (K) R space and (L) k space, respectively.

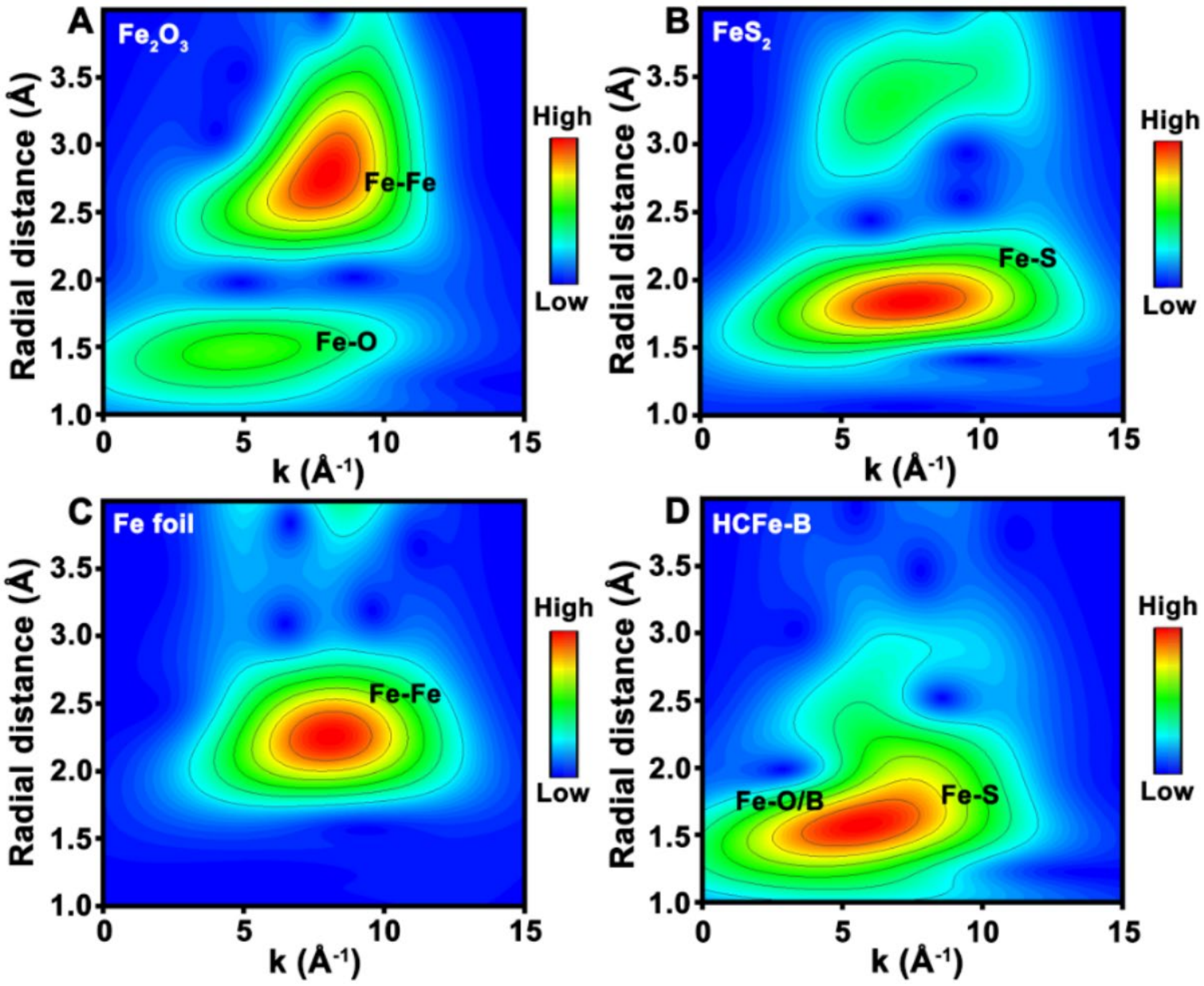

Fig S4 2D WT images of the Fe K-edge from (A) Fe_2O_3, (B) FeS_2, (C) Fe-foil and (D) HCFe-B nanozyme.

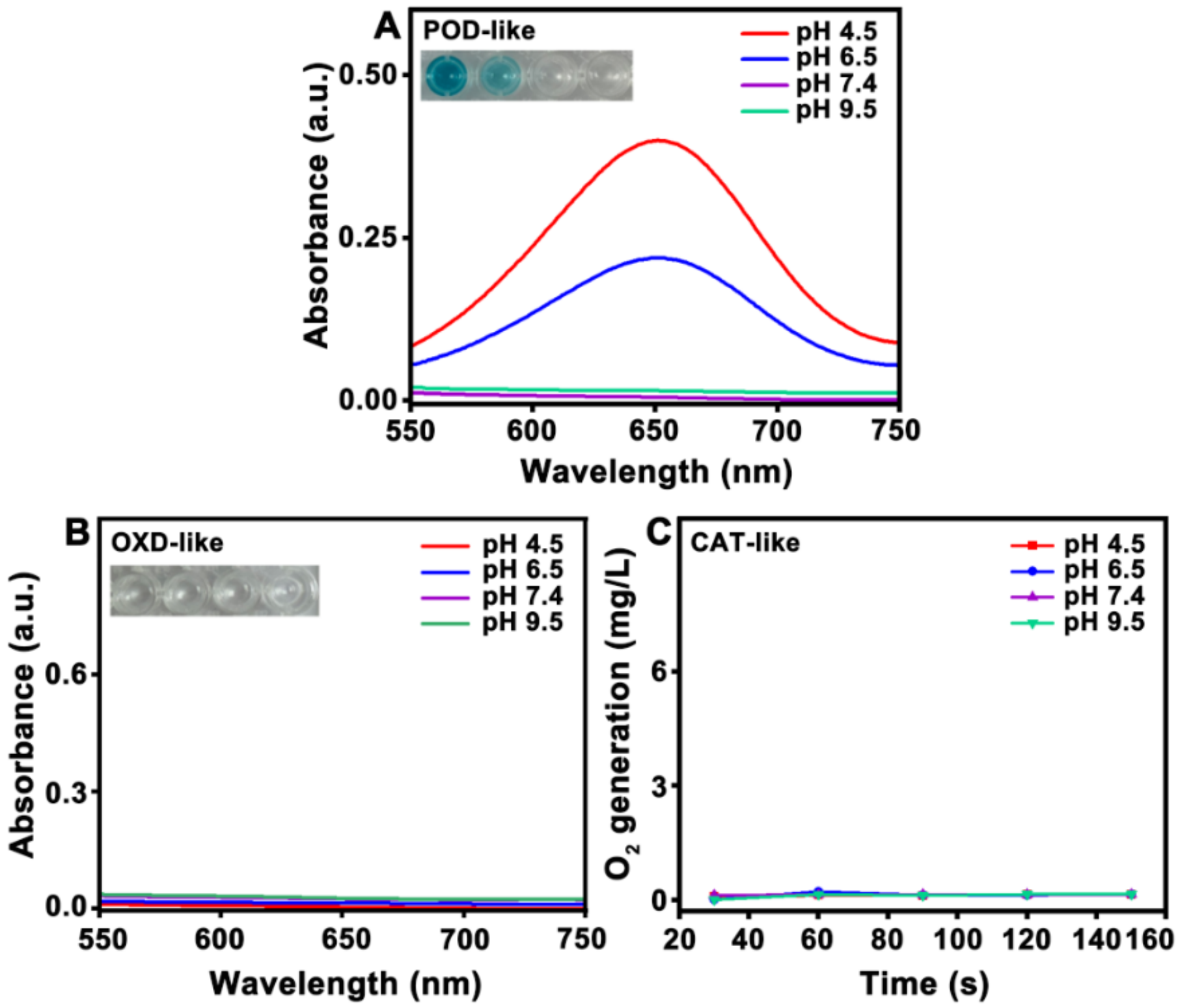

Fig S5 (A) POD, (B) OXD and (C) CAT catalytic activity of HCFe-B at different pH values.

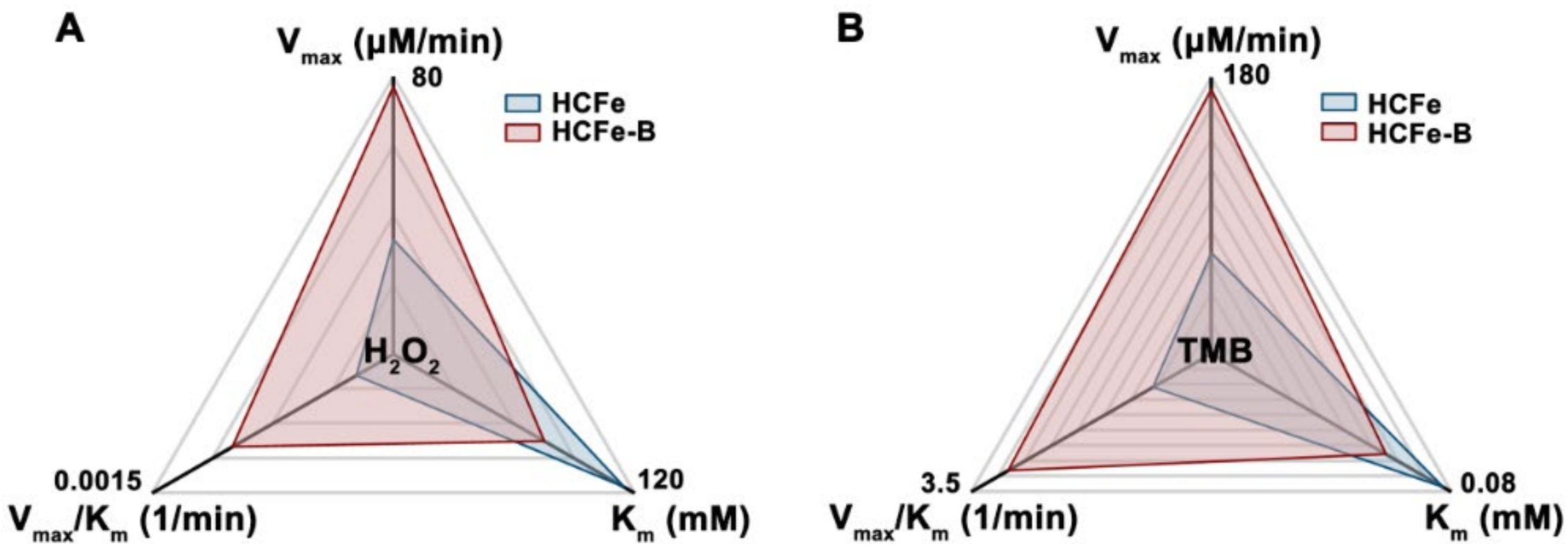

Fig S6 Kinetics parameter comparison for HCFe and HCFe-B.

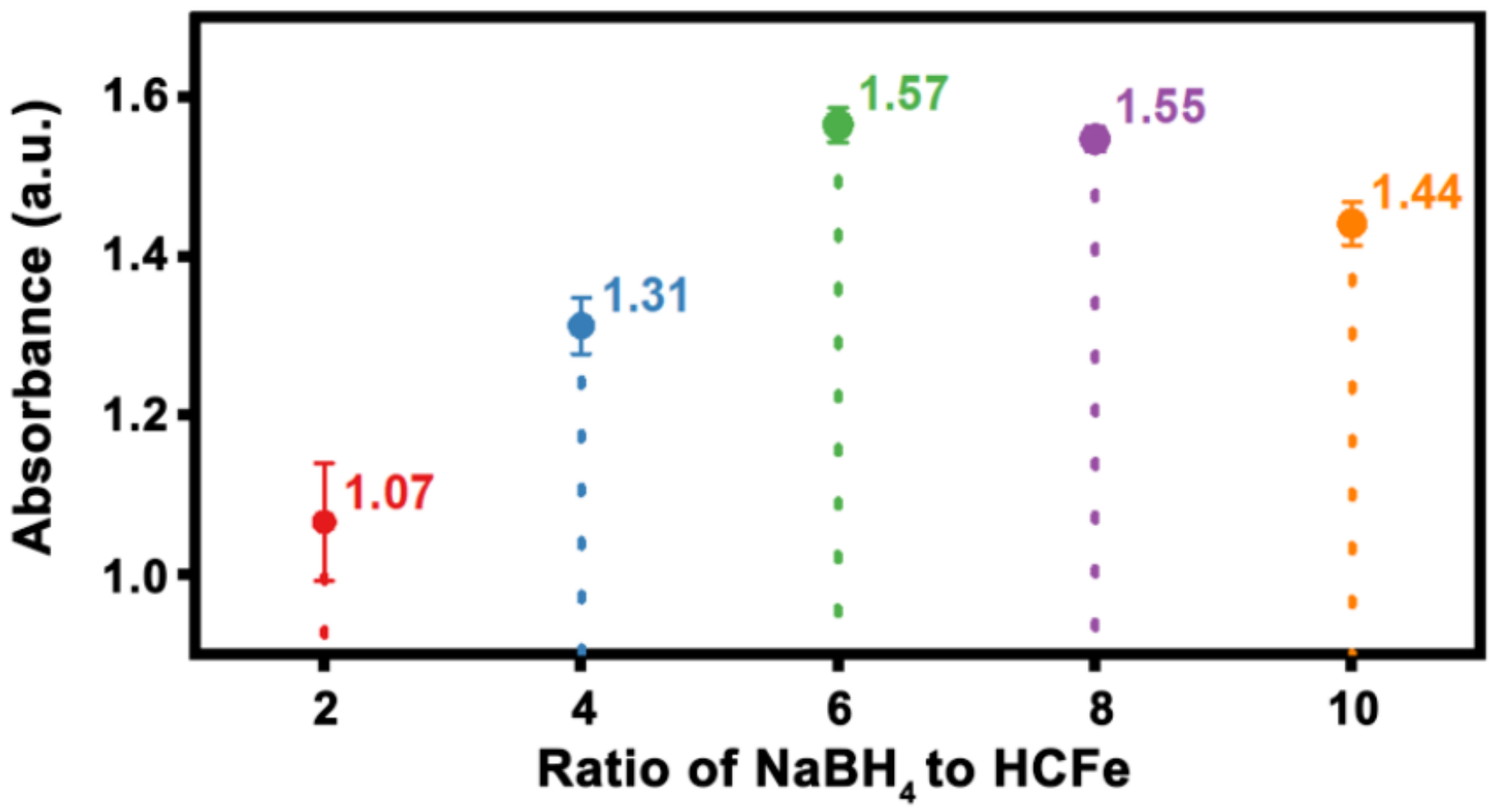

Fig S7 Optimization of B doping ratio.

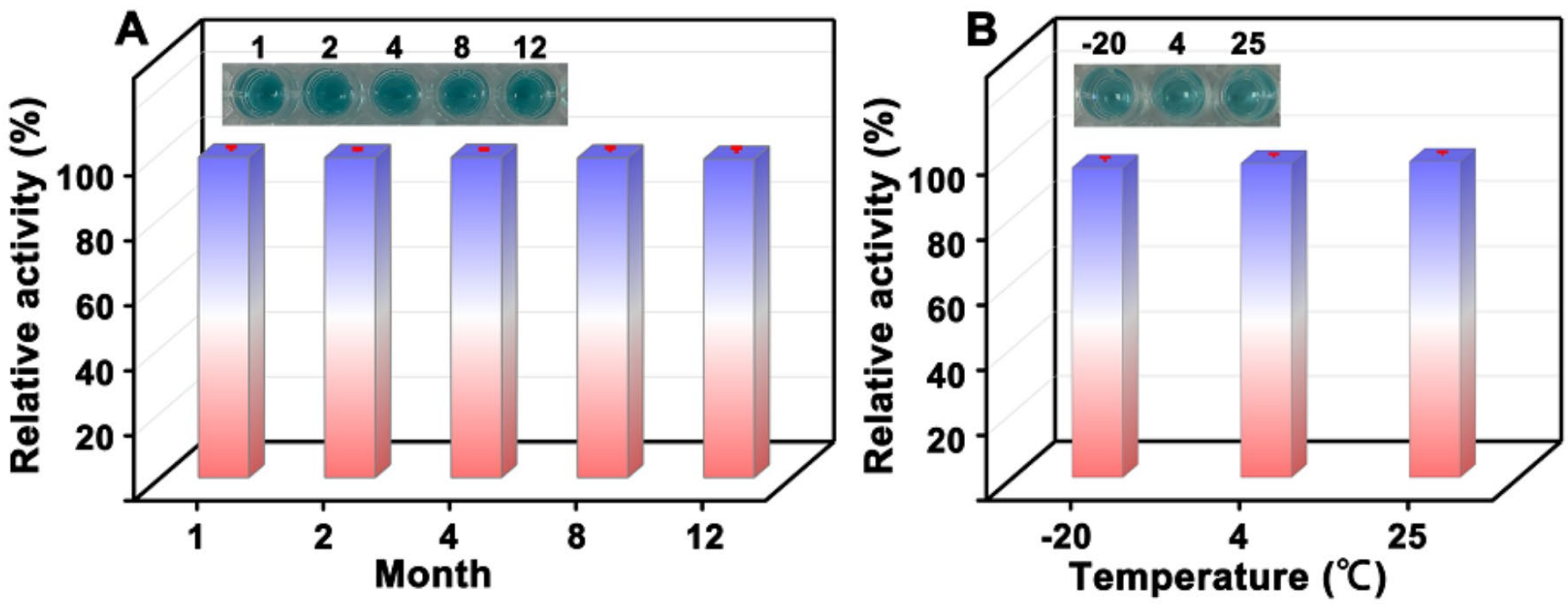

Fig S8 Stability of the catalytic activity of HCFe-B.

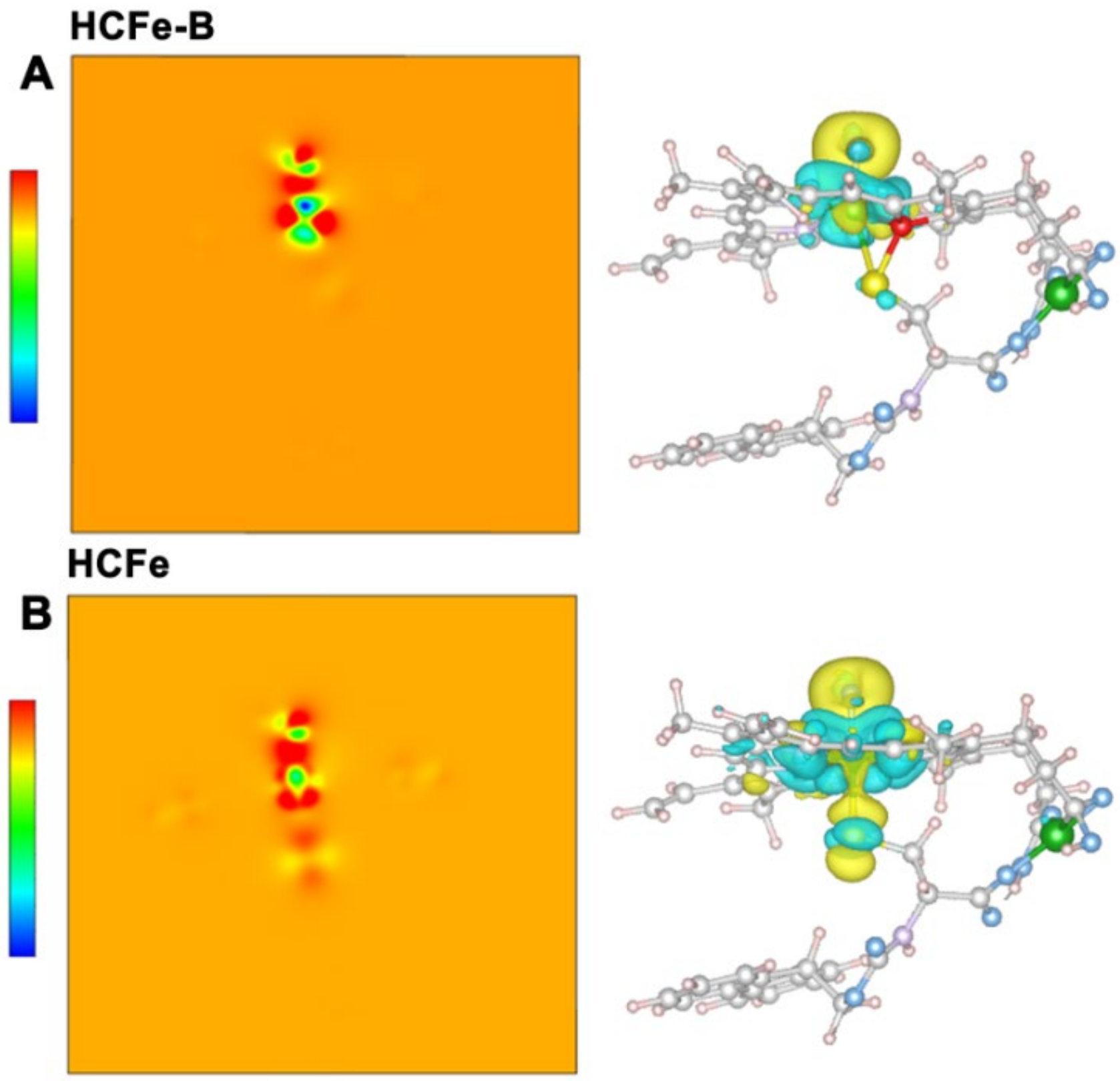

Fig S9 The charge density differences of (A) HCFe-B and (B) HCFe, respectively. (Isovalue: 0.002)

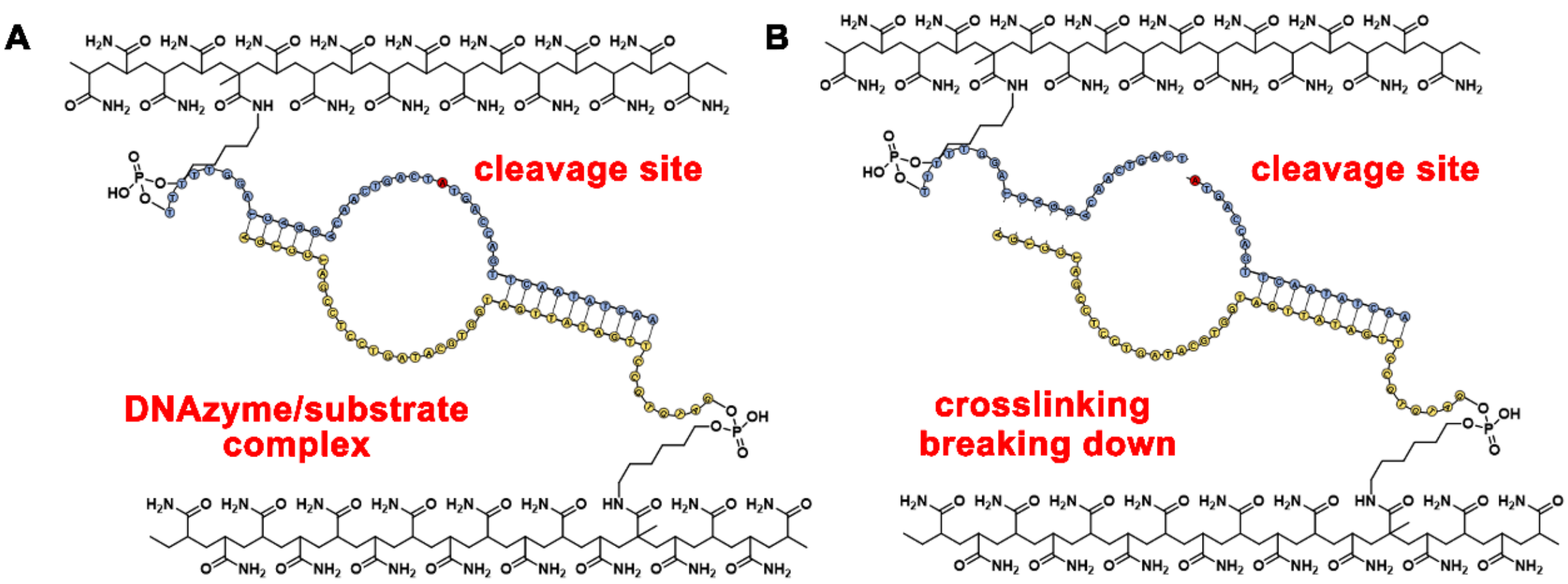

Fig S10 Chemical structure of the polymeric matrix showing an acrydite DNAzyme/substrate crosslink (A) before and (B) after being cleavage by target bacteria.

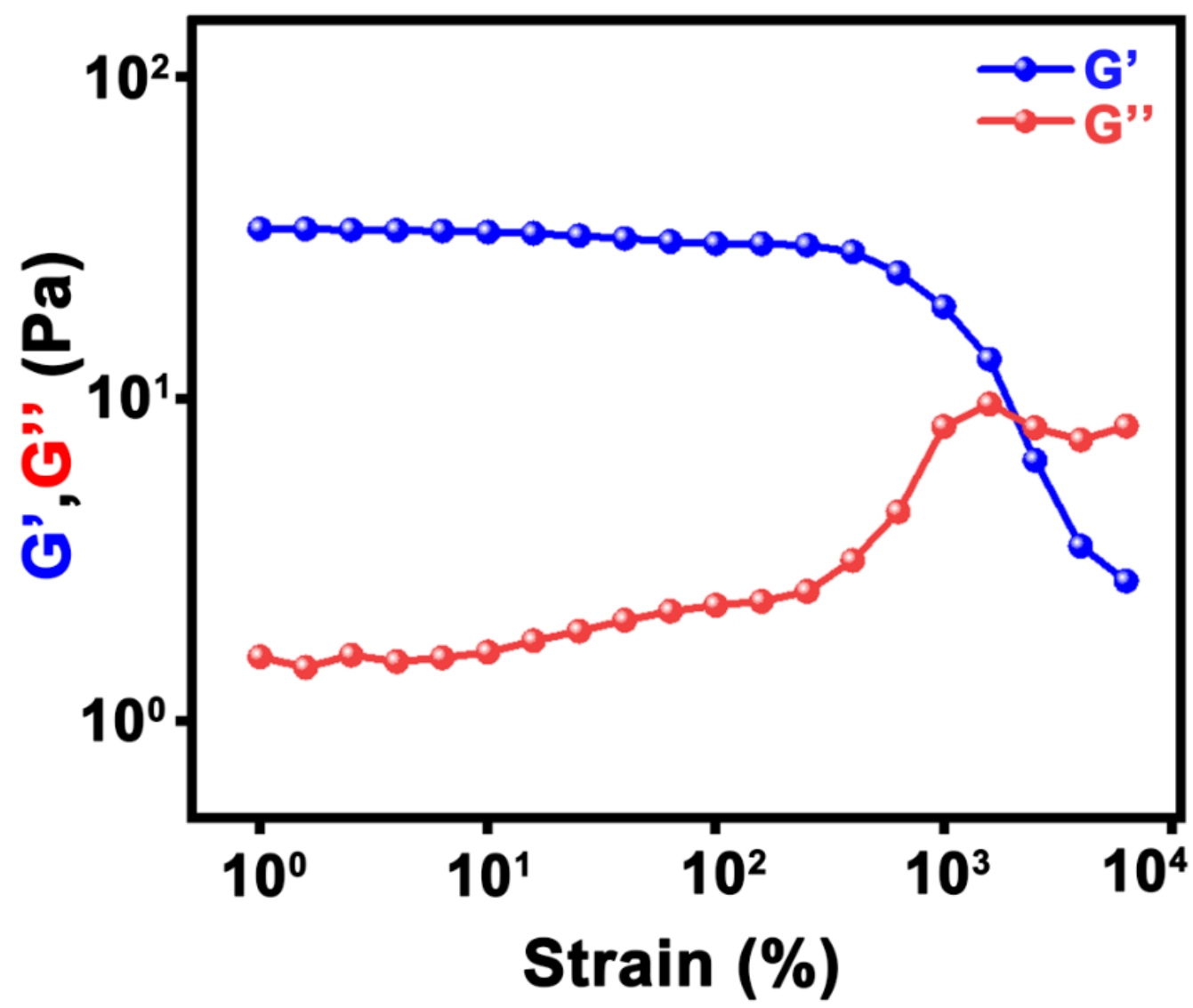

Fig S11 Rheology result of a strain sweep performed at a fixed frequency of 1 Hz at 25 °C.

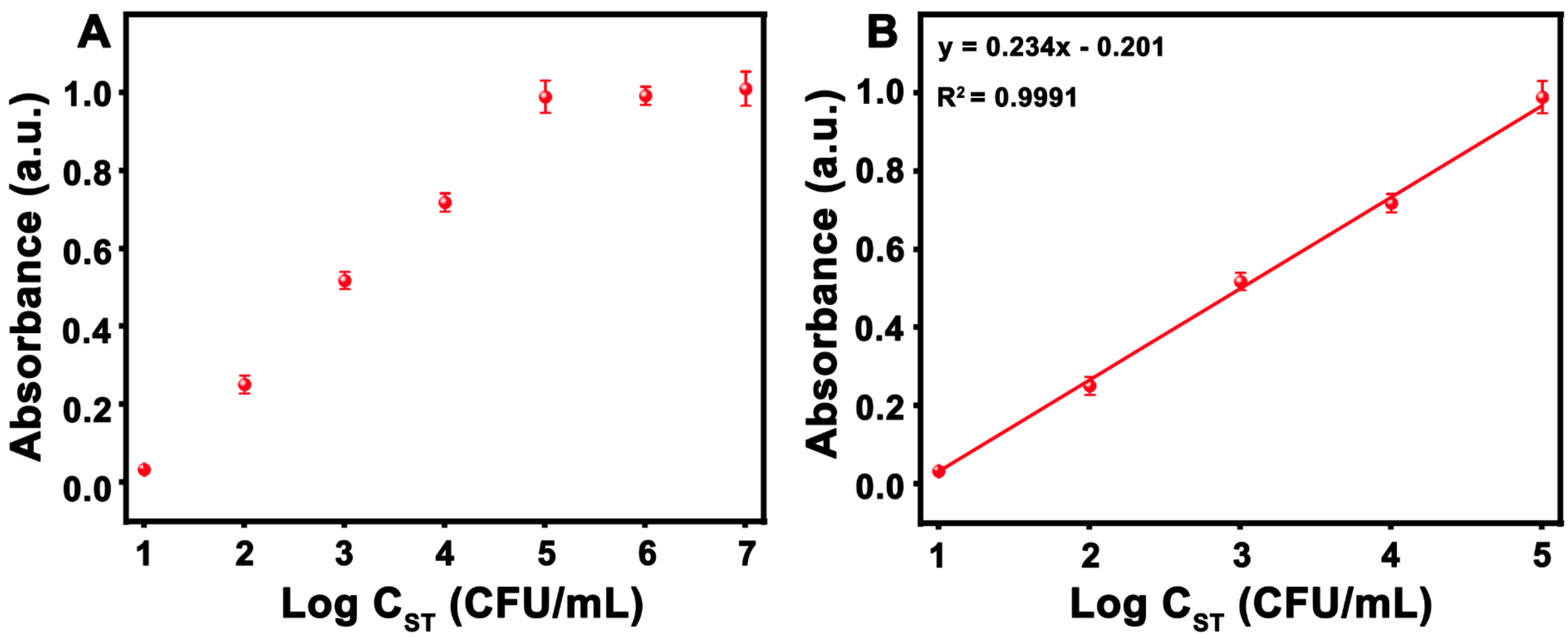

Fig S12 (A) Plot of absorbance versus ST concentration. Error bars represent the standard deviation (n = 3). (B) Linear correlation between absorbance and the logarithmic value of ST concentration over the range of 10^1 to 10^5 CFU/mL.

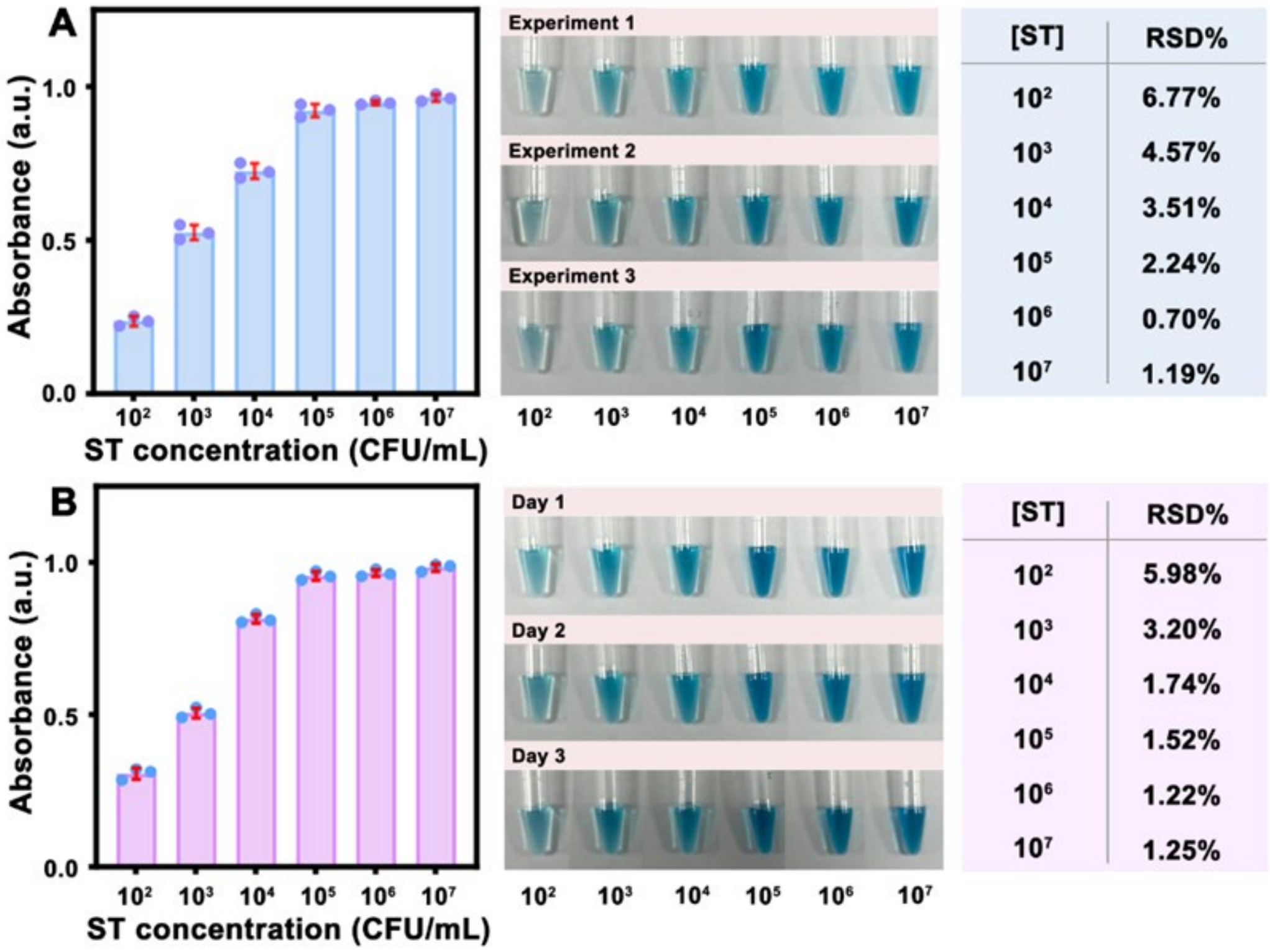

Fig S13 (A) Intra-day reliability test. Left: Absorbance responses at ST concentrations ranging from 10^2 to 10^7 CFU/mL (n=3). Middle: Photographs of the colorimetric reactions. Right: Corresponding relative standard deviations (RSD %) for three independent intra-day experiments. (B) Inter-day reproducibility test over three consecutive days. Left: Absorbance responses at ST concentrations ranging from 10^2 to 10^7 CFU/mL (n=3). Middle: Photographs of the colorimetric reactions. Right: RSD % values across Day 1, Day 2 and Day 3. The low RSDs indicate excellent stability and reproducibility of the proposed method.

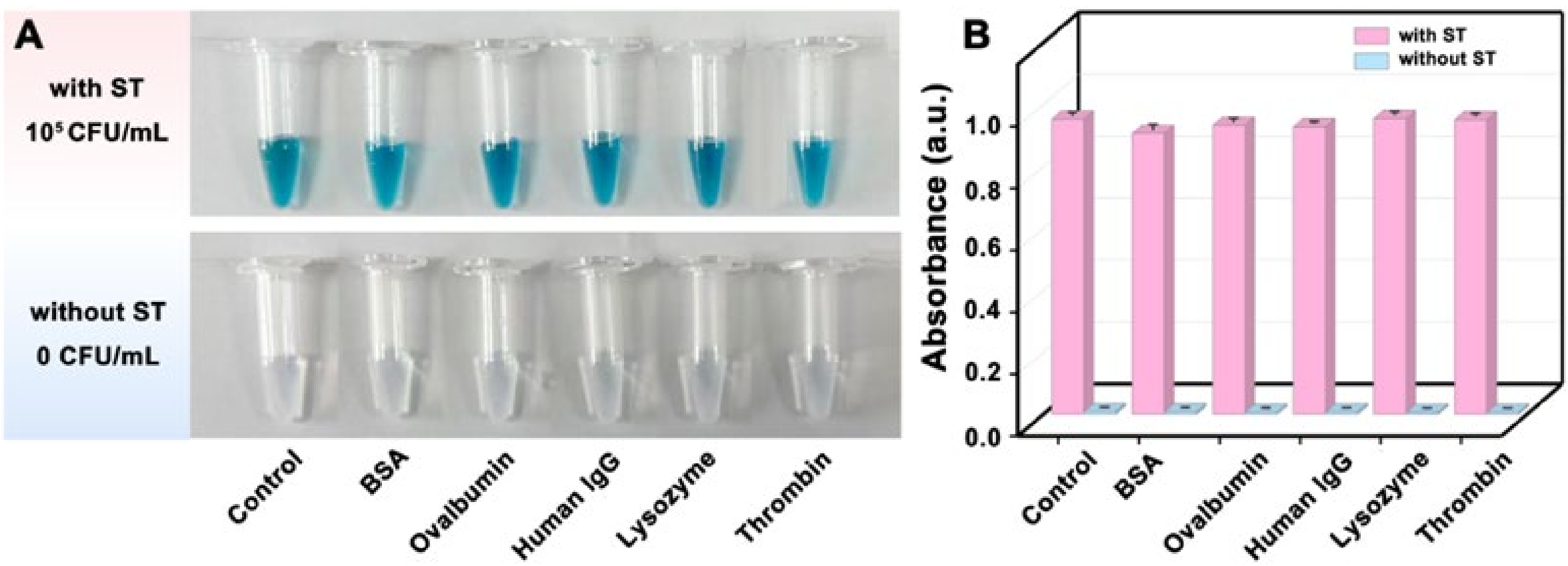

Fig S14 (A) Photographs showing the selectivity and anti-interference capability of the proposed sensing platform in the presence of various proteins. The assay was performed with ST at 10^5 CFU/mL (top row) and without ST (0 CFU/mL, bottom row) alongside different interfering substances, including BSA, ovalbumin, human IgG, lysozyme, and thrombin. (B) Corresponding absorbance values for the tested groups.

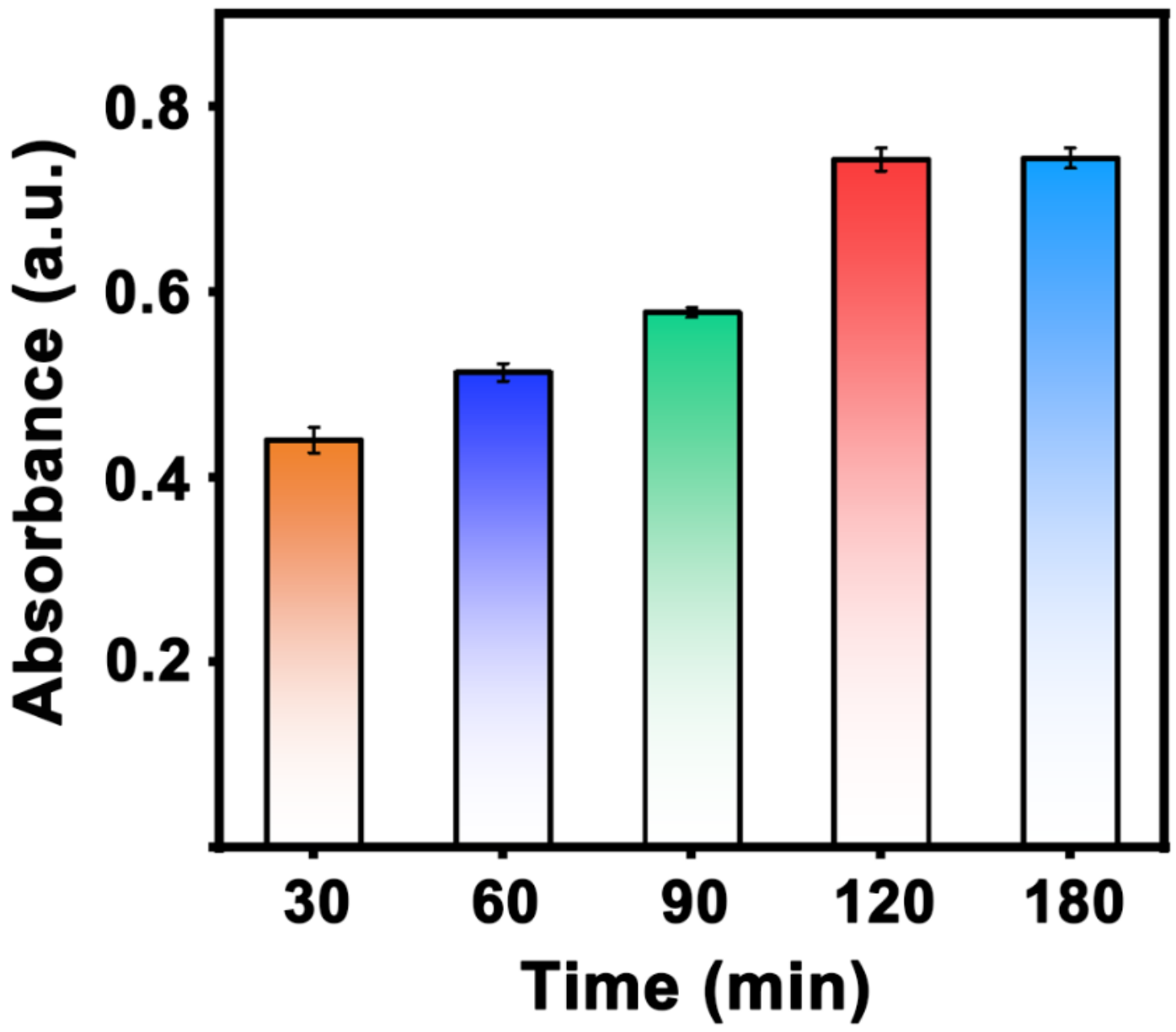

Fig S15 Optimization of the reaction time of the target-triggered DNAzyme hydrogel sensing platform for ST detection.

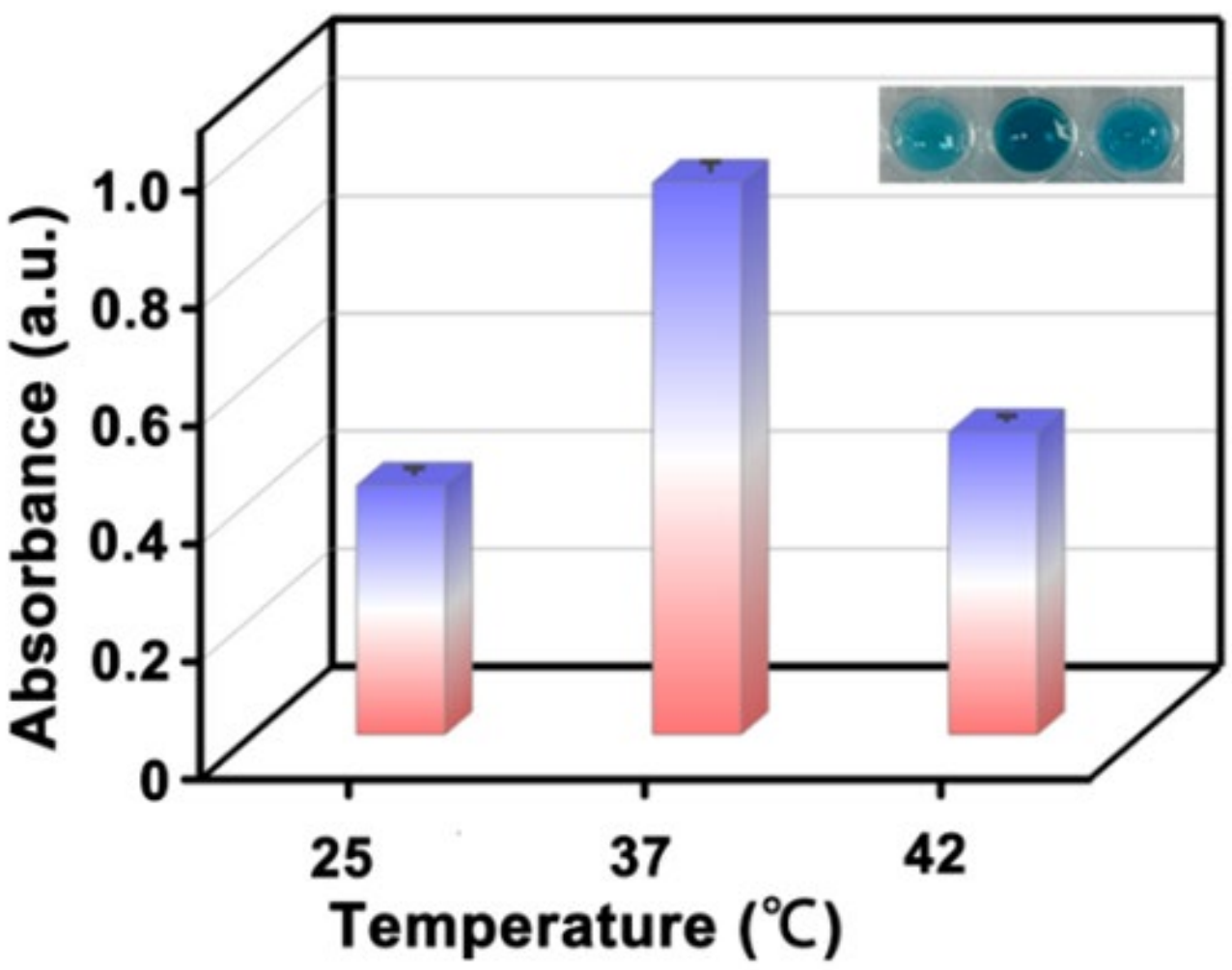

Fig S16 Effect of reaction temperature on the sensing performance for ST detection.

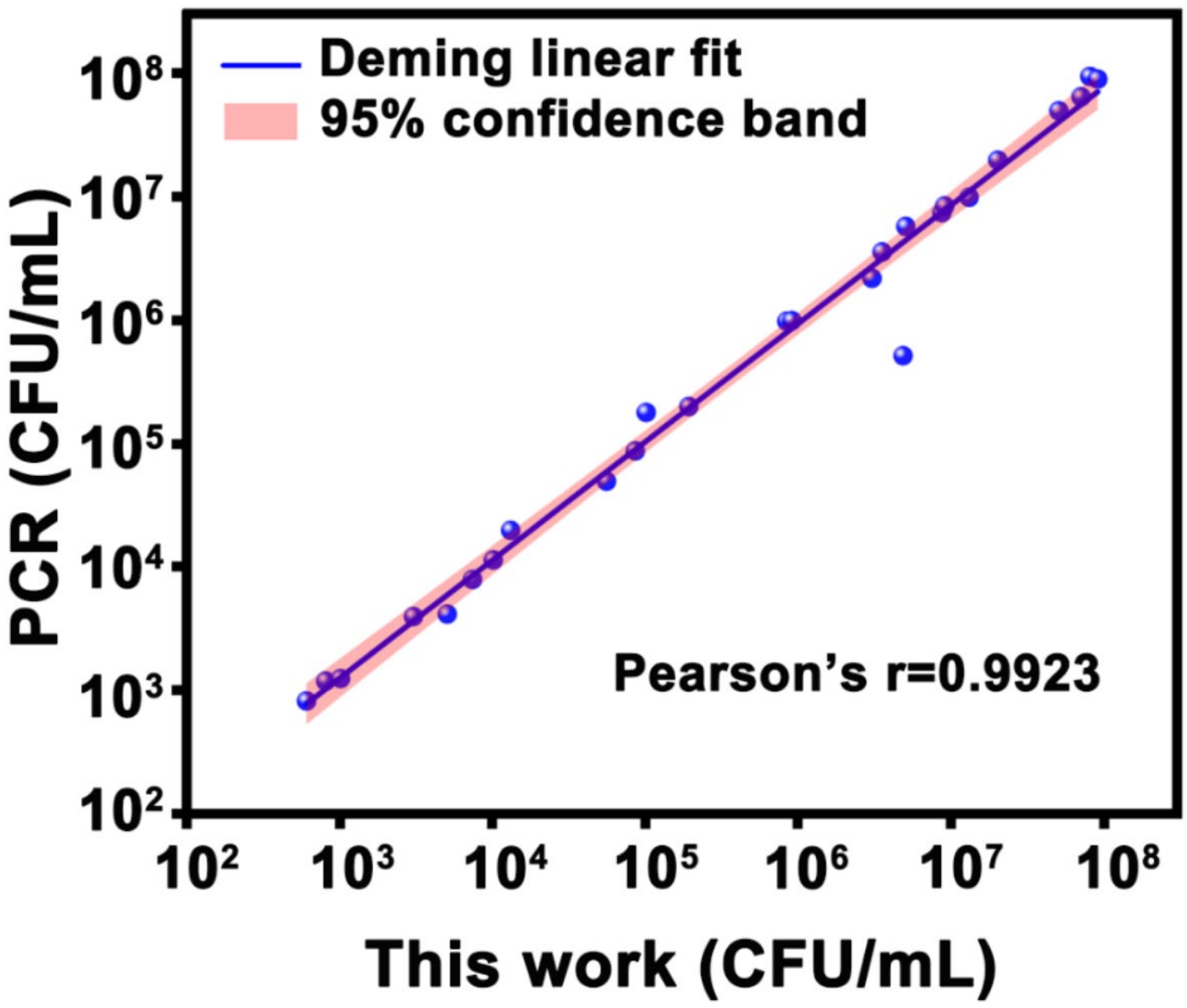

Fig S17 Correlation analysis of parallel detection results for ST using DNAzyme hydrogel colorimetric system versus the PCR method.
